# The Role of the Omega Subzone in Determining the Membership of a Protein in One of the Two Families of the LexA/Signal Peptidase-like Superfamily

**DOI:** 10.3390/ijms27052127

**Published:** 2026-02-25

**Authors:** Alexander I. Denesyuk, Konstantin Denessiouk, Mark S. Johnson, Vladimir N. Uversky

**Affiliations:** 1 Structural Bioinformatics Laboratory, Biochemistry, InFLAMES Research Flagship Center, Faculty of Science and Engineering, Åbo Akademi University, 20520 Turku, Finland; kdenessi@abo.fi (K.D.); mark.s.johnson@abo.fi (M.S.J.); 2 Department of Molecular Medicine, USF Health Byrd Alzheimer’s Research Institute, Morsani College of Medicine, University of South Florida, Tampa, FL 33612, USA

**Keywords:** signal peptidase, LexA endopeptidase, 3D structure, catalytic core, Omega subzone, protein family, structural marker

## Abstract

LexA/signal peptidase-like superfamily proteins are serine proteases that use the Ser-Lys catalytic dyad to carry out their biological functions. Here, we investigate the two known families of LexA/signal peptidase-like superfamily proteins, the type I signal peptidase and LexA endopeptidase domain-like, and describe the structural catalytic cores that govern the catalytic residues in these proteins. We show that the structural catalytic core of these proteins is a combination of two subzones, the NucBaseOmega and Omega. While the NucBaseOmega subzone is a pattern observed in all proteins of the studied superfamily, the Omega subzone in the type I signal peptidase family differs from that of the LexA endopeptidase domain-like family. Thus, the amino acids and 3D characteristics of the Omega subzone are a structural marker of the proteins belonging to a specific family.

## 1. Introduction

Earlier, we described the structural catalytic core (SCC) of the β-lactamase/transpeptidase-like superfamily of proteins [1]. From the point of view of function, the superfamily of β-lactamases/transpeptidases included bacterial enzymes that provided multi-resistance to β-lactam antibiotics such as penicillins [2]. Most proteins in that superfamily carried out their biological functions through the employment of a Ser-Lys catalytic dyad (InterPro entry: IPR012338 [3]). The SCC in those proteins had been defined as a minimal conserved structural organization that has common structural features and also incorporates the most important catalytic, functional and supporting residues. In [1] we also showed that the SCC in β-lactamases included residues of the Omega subzone, which was a specific substructure introduced by us, but which included fragments of the already known Ω-loop and the antiparallel bridge joining its terminal residues. From earlier studies, the Ω-loop is known to play an important role in molecular recognition and protein function [4,5], and the unrelated antiparallel bridge is one of the seven known patterns observed in the secondary structure of proteins, where between residues in positions “i” and “j” there are two hydrogen bonds that are characteristic of the β-structure [6].

The β-lactamase/transpeptidase-like proteins, structure and composition of their catalytic fragments, which are part of the described SCC, did stem from variations in the catalytic Ser/His/Asp triads in the overall group of serine proteases, and from the variety of emerging unconventional catalytic triad/dyad combinations in these enzymes [7,8]. In the current study, besides the β-lactamase/transpeptidase-like superfamily there is another unrelated superfamily, the LexA/signal peptidase-like superfamily (InterPro entry: IPR036286), whose enzymes also use the Ser-Lys catalytic dyad to carry out their function, and which originate from the same pool of unconventional serine proteases. In addition, there is a notable structural similarity between the proteins of both superfamilies in the organization of their oxyanion holes [7]. Additionally, the functional relationship between proteins of the two superfamilies is also highlighted by two facts: (1) β-lactam antibiotics induce the SOS response in *S. aureus*, which involves the activation of the LexA protein [9], and (2) bacterial signal peptidases are also inhibited by some β-lactam compounds [10].

Here, we decided to look at the SCC of LexA/signal peptidases and see how it relates to the SCC of β-lactamases/transpeptidases in order to evaluate the structural active site homology between the two superfamilies.

## 2. Results and Discussion

Members of the LexA/signal peptidase-like superfamily have an unconventional catalytic Ser-Lys dyad instead of the classical catalytic Ser-His-Asp triad. No other structural amino acid motifs have been identified for this superfamily of proteins. Consequently, it is possible that their structural catalytic cores (SCCs) predominantly use main chain interactions for their local substructure.

### 2.1. Creating a Dataset of the LexA/Signal Peptidase-like Superfamily Proteins

In the SCOP database [11], the LexA/signal peptidase-like superfamily includes two families: (1) the type I signal peptidase family with only one known protein, and (2) the LexA endopeptidase domain-like family with six different proteins. Additionally, the InterPro database [3] adds three other proteins to the type I signal peptidase family, totaling four known different proteins in that family; it also adds two other proteins to the LexA endopeptidase domain-like family, totaling eight different proteins in that family. All in all, the resulting set includes 12 different proteins in the LexA/signal peptidase-like superfamily. Each of these 12 different proteins has one or several known 3D structures in the Protein Data Bank (PDB [12]), where one structure each can be chosen as a “protein representative”. These structures are summarized in Table 1. In addition to protein representatives, from the InterPro database (IPR036286 and IPR039418) [3], we took the “family representative” structure for the type I signal peptidase family (one structure), and another structure as the family representative for the LexA endopeptidase domain-like family. These two structures are the signal peptidase I protein (LEP_ECOLI [13]; PDB ID: 1B12; R = 1.95 Å, [10]) and the lambda repressor CI (RPC1_LAMBD [13]; PDB ID: 1F39; R = 1.90 Å, [14]) (Table 1). Supporting the choice, PDB ID: 1B12 is also defined as the representative structure (SCOP ID: 4002992) of the respective protein family by SCOP [11]. The signal peptidase I protein (PDB ID: 1B12) is a hydrolase, which cleaves the leader peptide from a variety of secreted proteins [15], and the lambda repressor CI (PDB ID: 1F39) is the master regulator of the bacteriophage lambda life cycle. It is involved in the global SOS response to DNA damage, where lambda repressor CI is self-cleaved [14,16,17].

In addition to these 12 structures, the 3D structure of the signal peptidase IB from *S. aureus* (LEP_STAAC, PDB ID: 4WVJ, and R = 1.95 Å) [18] was used to study role of SCC in peptide–ligand interaction. For conformational analysis of SCC during the self-cleavage of the LexA endopeptidase domain-like family proteins, six sets of coordinates were used: RPC1_LAMBD (PDB ID: 8GMU, R = 2.78 Å) [19], LEXA_PSEAI (PDB ID: 8S7G, R = 3.43 Å) [20], LEXA_ECOLI (PDB IDs: 1JHE, R = 2.50 Å; 8GMS, R = 3.31 Å and 8TRG, R = 2.93 Å) [19,21,22] and UMUD_ECOLI (PDB ID: 8GMT, R = 3.31 Å) [19]. The final result was a set of 19 analyzed 3D structures. This list of 19 representative 3D structures contains all the currently available different proteins of the LexA/signal peptidase-like superfamily and their complexes with various peptides or ligands that reside in the active site.

**Table 1:** Structural catalytic core (SCC) in 12 LexA/signal peptidase-like superfamily representative proteins.

N	PDB ID	R (Å)	Protein	Nuc hexapeptide	Omega subzone	Cross-sheet ladder	Ref.
Superfamily: LexA/signal peptidase-like							
Family: Type I signal peptidase							
1	1B12_A	1.95	LEP_ECOLI	85 QIPSG**S** 90	272 GDNRDNSAD 280	82 EPF 84; 101 ILVE 104;294 GRATA 298;130 IVVF 133;143 YI**K**R 146;270 MM 271; S 281	[10]
2	4WVI_A	1.90	LEP_STAAC	382 TIKGE**S** 387	497 GDNREVSKD 505	379 TPY 381;398 VAVN 401;519 GKVS– 522;415 VVVF 418;426 YV**K**R 429;495 VL 496; S 506	[18]
3	4N31_A	2.20	R9TES9_STRPY	43 IINTND 48	140 NDYREERLD 148	40 GVM 42; 59 VLYY 62; 162 GKIST 166; 73 VVVY 76; 83 KVGR 86; 138 IL 139; S 149	[23]
4	7P2P_A	4.90	SC11A_HUMAN	51 VVLSG**S** 56	115 GDNNA--VD 121	48 PIV 50; 67 LFLT 70; 140 GRARG 144; 82 IVVF 85; 94 IV**H**R 97; 113 TK 114; D 122	[24]
Family: LexA endopeptidase domain-like							
5	1F39_A	1.90	RPC1_LAMBD	144 EVEGN**S** 149	205 PLNPQ–YPM 212	141 FWL 143;166 ILVD 169;223 GKVIA 227;179 FCIA 182;190 TF**K**K 193;203 LQ 204; I 213	[14]
6	2HNF_A	1.80	Q7B004_ECOLX	144 EVEGN**S** 149	205 PLNPQ–YPM 212	141 FWL 143;166 ILVD 169;223 GKVIA 227;179 FCIA 182;190 TFAK 193;203 LQ 204; I 213	[25]
7	3K2Z_A	1.37	LEXA_THEMA	114 KVKGE**S** 119	169 PANRE–MSS 176	111 FLL 113;131 VLVR 133;188 GKVVG 192;144 IVAA 147;154 TLAK 157;167 LR 168; M 177	[26]
8	6A2Q_A	1.48	LEXA_MYCTU	155 KVIGD**S** 160	210 PHNPA–FDP 217	152 FLL 154;172 VVVR 175;227 GKVVT 231;185 IVAA 188;195 TV**K**T 198;208 LM 209; I 218	[27]
9	8B0V_A	1.70	LEXA_PSEAI	120 RVRGM**S** 125	175 AENPE–FAP 182	117 YLL 119;137 LAVH 140;196 GLSVG 200;150 VVVA 153;160 TV**K**R 163;173 LL 174; I 183	[20]
10	1JHF_A	1.80	LEXA_ECOLI	114 RVSGM**S** 119	169 PENSE–FKP 176	111 FLL 113;131 LAVH 134;190 GLAVG 194;144 VVVA 147;154 TV**K**R 157;167 LL 168; I 177	[21]
11	1UMU_A	2.50	UMUD_ECOLI	55 KASGD**S** 60	109 PMNSA–YSP 116	52 YFV 54; 72 LIVD 75; 129 GVVIH 133; 85 IVIA 88; 95 TV**K**K 98; 107 LI 108; I 117	[28]
12	2FJR_A	1.95	RPC1_BP186	122 RSE---124	166 GG----KVP 170	119 MAI 121;128 YFVD 131;182 GRVVG 186;140 LWLV 143;150 SIRE 153;164 VA 165; F 171	[29]

Сatalytic base residues are shown in bold.

### 2.2. The Type I Signal Peptidase Family: SCC in the Signal Peptidase I from E. coli

#### 2.2.1. The NucBaseOmega Subzone Around the Ser-Lys Catalytic Dyad

**Figure 1:**
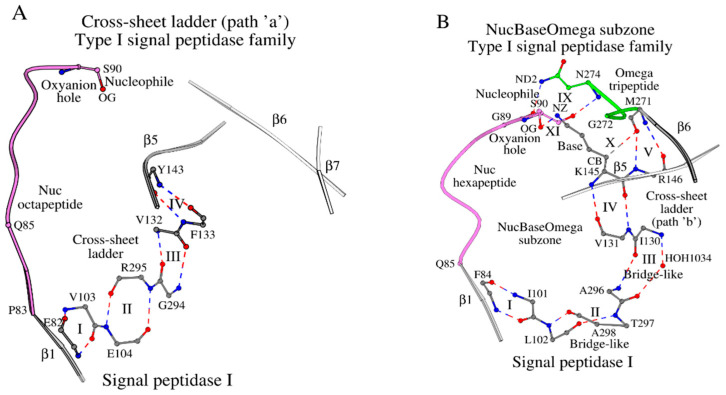
Plots (**A**,**B**) show paths “a” and “b” of the cross-sheet ladder in the type I signal peptidases, respectively. (**A**) Four antiparallel bridges, which are marked with numbers I–IV, form path “a” of the cross-sheet ladder. The Nuc octapeptide is the peptide Pro83-Ser90 joining the catalytic nucleophile with the cross-sheet ladder via the β1 β-strand. (**B**) The NucBaseOmega subzone includes the Nuc hexapeptide (Gln85-Ser90), three antiparallel bridges (steps I, IV and V of the cross-sheet ladder), two antiparallel bridge-like interactions (steps II and III of the cross-sheet ladder), and the Omega tripeptide (Gly272-Asn274). Interactions marked as I through XI are described in Tables S2 and S3.

The structure of LEP_ECOLI has a mainly β-sheet protein fold, consisting of two large antiparallel β-sheet domains, I and II [10]. The β-sheet of domain I consists of seven β-strands, β1 through β7, and incorporates the catalytic base, Lys145, the catalytic nucleophile, Ser90, and the oxyanion atom N/Ser90, where the catalytic nucleophile lies in the loop following strand β1 (Tyr81-Gln85), and the catalytic base lies within strand β5 (Asp142-Gly149). The β-sheet of domain I twists significantly into a barrel-like structure, so that β-strands β1 and β5 approach each other and the catalytic base, Lys145, and nucleophile, Ser90, approach in space, which is required by protein function. Strands β6 and β7 turn away from the β1–β5 barrel-like structure, and the β1 and β5 strands are locked by two separate paths (two cross-sheet ladders, “a” and “b”) made of consecutive antiparallel bridges [6] (ladder steps), described in the Introduction. The cross-sheet ladder “a” starts with Glu82 (β1) and ends with Tyr143 (β5) (Figure 1A; Table S1), while the cross-sheet ladder “b” starts with Phe84 (β1) and ends with Arg146 (β5) (Figure 1B; Table S1). The steps of the cross-sheet ladders “a” and “b” are summarized in Table S2. The cross-sheet ladder “a” has four steps, which are all antiparallel bridges, I–IV (Figure 1A), according to criteria given in [6]. The cross-sheet ladder “b” also has four steps, I–IV (Figure 1B), but only two of those, I and IV, are typical antiparallel bridges according to criteria given in [6], while steps II and III are antiparallel bridge-like interactions, where antiparallel bridge-like interactions differ from antiparallel bridges by appearance of additional intermediate elements, such as additional interactions or intermediate water molecules between contacting residues.

**Figure 2:**
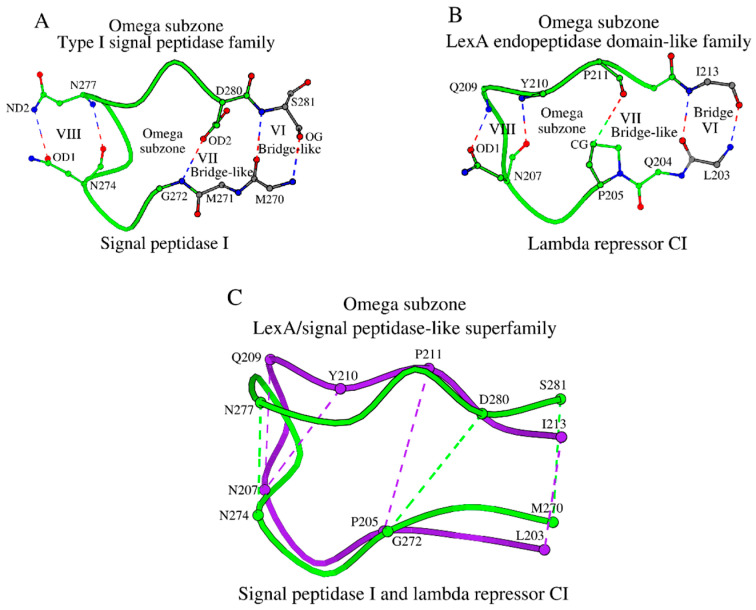
Plots (**A**–**C**) show Omega subzone in the type I signal peptidase family, the LexA endopeptidase domain-like family, and their superposition. (**A**) The representative structure is the signal peptidase I. (**B**) The lambda repressor CI. (**C**) Superposition of these two subzones. The Omega subzones of signal peptidase I and lambda repressor CI are shown in green and purple, respectively. Interactions marked as VI, VII and VIII are described in Tables S2 and S3.

In addition to steps I-IV, the cross-sheet ladder “b” has a joint antiparallel bridge V, which connects strands β5 and β6 (Figure 1B). The summary of amino acid segments (21 residues), which incorporate all interacting amino acids from the cross-sheet ladders “a” and “b”, are shown in Table 1 and Table S1 (“Cross-sheet ladder”). Met271, which resides in strand β6 in LEP_ECOLI and forms bridge V (Figure 1B), does also interact with the catalytic base Lys145 via a weak hydrogen bond (X in Figure 1B; column X in Table S3), and borders the tripeptide Gly272-Asn274 (Omega tripeptide), which coordinates positioning of the Ser90 nucleophile via a conventional hydrogen bond (IX in Figure 1B; column IX in Table S3). Finally, the Ser-Lys catalytic dyad forms a canonical functional interaction between these two residues (shown as XI in Figure 1B). As a result, the interlocked NucBaseOmega subzone around the Ser-Lys catalytic dyad is formed, which includes: (1) the β1-loop-Nucleophile peptide (shown as Nuc octapeptide in Figure 1A or Nuc hexapeptide in Figure 1B); (2) the “Cross-sheet ladder (β1–β5)/Catalytic Base” local structure, joined by the antiparallel bridges; and (3) the Omega tripeptide. The choice of the Omega tripeptide and not the entire Omega subzone here is due to its functional role of coordinating the catalytic nucleophile residue. The NucBaseOmega subzone can be traced in all proteins of the type I signal peptidase family.

#### 2.2.2. The Omega Subzone in the Type I Signal Peptidase Family Proteins

The cross-sheet ladder of the NucBaseOmega subzone in type I signal peptidases involves amino acids from β-strands β1–β6, which are linked to each other via nine antiparallel bridge and bridge-like interactions: four interactions in ladder “a”, four interactions in ladder “b”, and one interaction V between Arg146 and Met271 (Figure 1A,B). However, the adjacent Met270 from strand β6 forms a reinforcing antiparallel bridge-like interaction to Ser281 (shown as VI in Figure 2A), which can also be observed in all type I signal peptidases, and thus Ser281 and its respective residues in the other type I signal peptidases were also added to the total cross-sheet ladder connection (Table 1 and Tables S1 and S2). The existence of interaction VI indicates the existence of a separate, locked structural Omega subzone (Figure 2A). The conformation of the Omega subzone is stabilized by several hydrogen bonds (shown as bridges VI, VII and VIII in Figure 2A, and in columns VI, VII and VIII in Tables S2 and S3). Here, residue Asn274 not only participates in the interaction with the catalytic nucleophile (Figure 1B), but also in the interactions with Asn277 (Figure 2A), fixing the conformation of the Omega subzone. As discussed below, this part of the Omega subzone participates in ligand binding by type I signal peptidases.

#### 2.2.3. The Structural Catalytic Core (SCC) of the Type I Signal Peptidases

**Figure 3:**
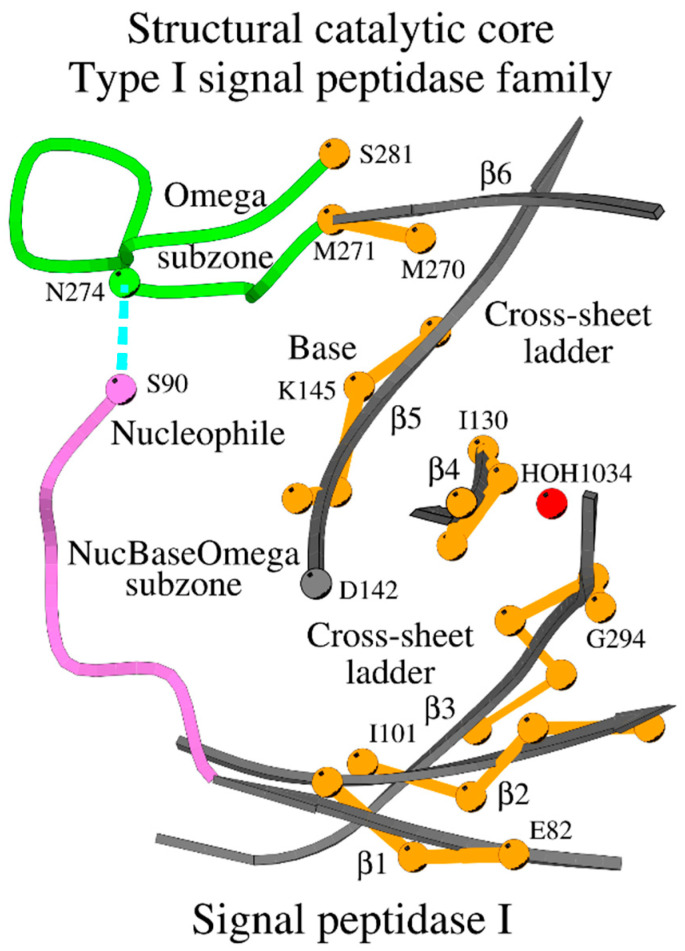
Overview of the B-type structural catalytic core or B-SCC of the type I signal peptidase-like family. The Nuc-peptide is shown in purple, the residues of the cross-sheet ladder in orange, and the Omega subzone in green. The dotted line shows the contact between the nucleophile and the conserved asparagine of the Omega subzone. Several amino acids are marked for reference purposes.

The structural catalytic core (SCC) of the type I signal peptidases incorporates the NucBaseOmega and Omega subzones (Figure 3). Figure 3 shows how residues of the cross-sheet ladder function as a spine, supported by the β-sheet, for the key functional loops and residues. We will further refer to this type of SCC as the B-type SCC or B-SCC.

### 2.3. The Type I Signal Peptidase Family: SCC in the Other Proteins

Table 1 contains four representative proteins of the type I signal peptidase family, all with the B-type SCC. Below, we will compare the B-SCC in signal peptidase I from *E. coli* and in the other three representative structures of the family.

#### 2.3.1. SCC in the Signal Peptidase IB from *S. aureus*

The comparison of B-SCCs in LEP_ECOLI and LEP_STAAC [18] showed only three minor differences. The first difference lies in the structure of the cross-sheet ladder, where the Gly294-Ala298 in LEP_ECOLI is one amino acid longer than Gly519-Ser522 in LEP_STAAC (Table 1 and Table S1). Instead, an additional water molecule HOH814 is present in LEP_STAAC in the structure of the bridge II (see column II in Table S2). The second difference is the replacement of Asn277 (LEP_ECOLI) with Val502 (LEP_STAAC) in the turn segment of the Omega subzone, which may lead to higher flexibility of the Omega tripeptide and the overall Omega subzone (see interaction VIII in Figure 2A, and in Table 1 and Tables S1 and S3). Finally, the last difference is the presence of a water molecule mediator HOH857 between the residues of the catalytic dyad (as opposed to the direct interaction (see XI in Figure 1B and in Table S3).

#### 2.3.2. SCC in the Signal Peptidase-like Protein from *S. pyogenes*

This signal peptidase-like protein (R9TES9_STRPY) lacks the typical catalytic Ser and Lys residues characteristic of type I signal peptidases (Table 1 and Table S1). They are replaced by Asp48 and Gly85 residues, respectively, which play no part in function [23]. Additionally, there are minor changes in composition of the Omega subzone: Asn274 (LEP_ECOLI) → Tyr142 (R9TES9_STRPY) and Asn277 (LEP_ECOLI) → Glu145 (R9TES9_STRPY). All these changes do not affect the relative positioning of the Nuc peptide, the Omega subzone, and the cross-sheet ladder, i.e., they have no effect on the structural arrangement of the B-SCC, and thus it remains conserved between LEP_ECOLI and R9TES9_STRPY (Table S3).

#### 2.3.3. SCC in the Human Signal Peptidase Complex Catalytic Subunit SEC11A

The last representative structure of the type I signal peptidase family of proteins is the human signal peptidase complex catalytic subunit (SC11A_HUMAN) determined by the electron microscopy method, with a relatively poor resolution of R = 4.90 Å [24]. SC11A_HUMAN exists in structures of two paralogs: paralog A (PDB ID: 7P2P) and paralog C (PDB ID: 7P2Q). The two structures are indistinguishable at 5 Å resolution, and paralog A was chosen for further analysis because of a more stable conformation within the complex [24].

The catalytic dyads of the human (SC11A_HUMAN) and bacterial (LEP_ECOLI) signal peptidases differ in the type of amino acid used as the catalytic base: histidine versus lysine, respectively (Table 1). Also, there is a reduction in length of the Omega subzone. Regardless these changes, and also taking into account the poor resolution of 5Å, we can safely say that all the geometric parameters between the bacterial and human signal peptidases’ B-SCCs are conserved (Tables S1–S3).

### 2.4. The Role of SCC in Peptide Ligand Binding in Type I Signal Peptidases

**Figure 4:**
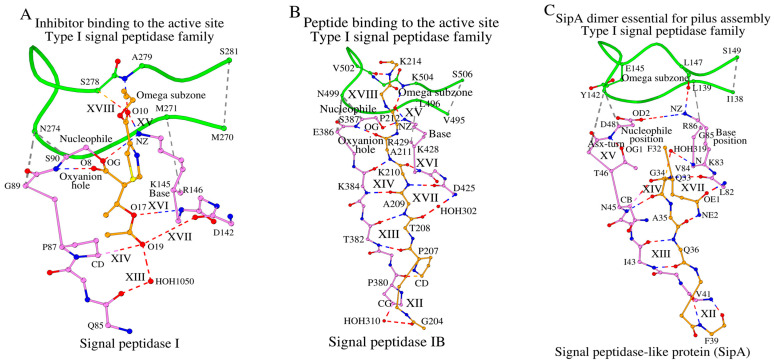
Three different types of sandwich-like interactions between proteins of the type I signal peptidase-like family and their peptide ligands. (**A**) shows signal peptidase I, (**B**) shows signal peptidase IB and (**C**) shows SipA protein. Ligands are shown in yellow, and the Omega zone in green. Interactions marked as XII through XVIII are described in Table S4.

In the signal peptidase I from *E. coli* (LEP_ECOLI), the C-terminal segment of the Omega subzone participates in ligand binding, where Ser278 and Ala279 form hydrogen bonds with the ligand atoms, shown as XVIII in Figure 4A and described in column XVIII in Table S4. The catalytic base, nucleophile and the oxyanion hole interact with the central ligand atoms O8, OG and O10 as a part of protein function (Figure 4A). On the other side of the peptide ligand, in the C-terminal part, the O17 and O19 atoms are sandwiched between the Nuc-peptide and the cross-sheet ladder, and form interactions XIII, XIV, XVI and XVII (Figure 4A and Table S4).

In the signal peptidase IB from *S. aureus* (LEP_STAAC), the C-terminal residues of the Omega subzone, similar to LEP_ECOLI, are also used for ligand binding; antiparallel tripeptides Val502-Lys504 (from the protein Omega subzone) and Pro212_D-K214_D (from the ligand peptide) form hydrogen bonds with each other, and interaction XVIII is present (Figure 4B; Table S4). Also, similar to LEP_ECOLI, the NZ atom of the catalytic base, Lys428, interacts with atom O/Pro212_D of the ligand making interaction XV (Figure 4B; Table S4), and the “oxyanion hole” main-chain NH atom of Ser387 makes a hydrogen bond to the carbonyl oxygen atom of Ala211_D, which is the C-terminal alanine of the conserved Ala-Xaa-Ala motif, after which (except there is a following proline) the enzyme cleaves the substrate [18]. As also seen in LEP_ECOLI, the N-terminal part of the peptide substrate (Pro207-Lys210 in LEP_STAAC) is sandwiched between the Nuc-peptide (Thr379-Lys384 in LEP_STAAC) and the cross-sheet ladder (Asp425-Arg429 in LEP_STAAC) forming interactions XIII, XIV, XVI and XVII (Figure 4B; Table S4). Moreover, in this sandwich, the Thr382-Lys384 tripeptide from the Nuc-peptide, the Pro207-Lys210 tetrapeptide from the ligand and the Asp425-Val427 tripeptide from the cross-sheet ladder have the conformation of a three-stranded parallel β-sheet that interacts with each other by means of the acceptor–donor–acceptor (ADA) and donor–acceptor–donor (DAD) interaction patterns, as seen in the ligand binding of many unrelated protein complexes [30]. Because of this motif feature, we will refer to the Asp425-Val427 tripeptide, which interacts with the peptide ligand by the stretched β-sheet type ADA/DAD interactions XVI and XVII and precedes in sequence the catalytic base (Lys428 in LEP_STAAC), as the DAD_BASE_ motif tripeptide.

In the signal peptidase homolog SipA from *S. pyogenes* (R9TES9_STRPY), the binding of the peptide ligand is functionally different, because SipA has lost the catalytic dyad typical for signal peptidases and functions in the polymerization of pilin subunits [23]. If we compare the binding of ligand peptides in LEP_ECOLI (Figure 4A), LEP_STAAC (Figure 4B) and R9TES9_STRPY (Figure 4C), we can see several fundamental differences between R9TES9_STRPY and the canonical type I signal peptidases. Firstly, because of the functional differences between R9TES9_STRPY and the canonical type I signal peptidases, the Omega subzone in R9TES9_STRPY is not involved in ligand binding (Figure 4C). Then, unlike in LEP_STAAC (Figure 4B), the peptide ligand is sandwiched between the Nuc-peptide segment Phe39-Asp48 and the Base-peptide segment Leu82-Arg86 in an antiparallel manner, where Ile43-Asn45 of the Nuc-peptide interacts with the Gly34-Gln36 of the bound ligand in an antiparallel β-strand conformation (interactions XIII and XIV in Figure 4C, and columns XIII and XIV in Table S4). Finally, in the signal peptidases I and IB, the DAD_BASE_ motif tripeptide sandwiches the ligand chain with the Nuc-peptide from the other side, while in SipA the DAD_BASE_ motif interacts with the side chain group of a single amino acid, Gln33. Also, in R9TES9_STRPY the absence of the catalytic function is reinforced by blocking the oxyanion hole, N/Asp48, by the presence of an Asx-turn [31]. Plus, the structural absence of a lysine at the catalytic base position 85 is partially compensated by a lysine at position 83 (Figure 4C), which forms interactions with the residue at the catalytic nucleophile position Asp48, NZ/Lys83-OD2/Asp48, and with the Omega subzone, NZ/Lys83-O/Leu147.

### 2.5. SCCs in the Type I Signal Peptidase Family: Conclusions

In summary, through the structural comparison of the SCCs of the four type I signal peptidase family representative proteins—the signal peptidase I from *E. coli* (LEP_ECOLI), the signal peptidase IB from *S. aureus* (LEP_STAAC), the signal peptidase-like SipA from *S. pyogenes* (R9TES9_STRPY), and the human signal peptidase complex catalytic subunit SEC11A (SC11A_HUMAN)—we can conclude that bacterial and human signal peptidase SCCs are structurally similar, even though some differences remain, such as (1) missing residues and the respective presence of additional water molecules in some steps of the cross-sheet ladder in LEP_STAAC, and (2) the reduction in the length of the Omega subzone in SC11A_HUMAN.

We can also conclude that in LEP_ECOLI and LEP_STAAC, the binding of peptide ligands with the NucBaseOmega and Omega subzones is similar, while in R9TES9_STRPY the Omega subzone does not participate in peptide ligand binding, even though interactions between the peptide ligand and the NucBaseOmega subzone are similar in all three peptidases.

Finally, we can conclude that the three peptidases use the structural DAD_BASE_ motif tripeptide for binding the peptide ligand in a similar donor–acceptor–donor way. This tripeptide borders the position of the catalytic base and has the overall schematics of “N_(BASE residue-1)_–O_(BASE residue-3)_–N_(BASE residue-3)_”, where “N” and “O” designate main-chain nitrogen and oxygen atoms of the residues preceding the catalytic base by one and by three, respectively.

### 2.6. SCC in the LexA Endopeptidase Domain-like Family Proteins

Similar analyses of the SCC in the lambda repressor CI (RPC1_LAMBD) showed that there are no significant differences in the construction of the NucBaseOmega subzone in lambda repressor CI and the signal peptidase I proteins (Table 1 and Tables S1 and S2). The existing differences between the SCC of the two families lie in the structure of their Omega subzones (Figure 2A vs. Figure 2B,C; Table S3), where in lambda repressor CI it is one residue shorter, with a single Tyr210 instead of an Asn277-Ser278 dipeptide (Table 1). Such sequence difference shortening induces an Asx-motif [32] within interactions VIII in lambda repressor CI (Figure 2B). Additionally, within the same Omega subzones, in lambda repressor CI an internal strong hydrogen bond VII connecting their N- and C-ends is lost, and Pro205 makes a weaker interaction (Figure 2A vs. Figure 2B,C; Table S3). Due to the presence of the side-chain atoms of Pro205 between the N- and C-ends of the Omega subzone, the distance between them increases by 1.1 Å in lambda repressor CI compared to the signal peptidase I, which affects its interactions with the NucBaseOmega subzone and the catalytic nucleophile.

### 2.7. SCC in a Non-Canonical Repressor Protein CI

Of the other seven representative structures of the LexA endopeptidase domain-like family proteins, six have SCCs similar to the lambda repressor CI and only the repressor CI from Eganvirus ev186 (RPC1_BP186; PDB ID: 2FJR [29]) is different (Table 1). The RPC1_BP186 does not form a canonical active site due to the absence of the C-terminal half of the Nuc-peptide and the catalytic base. The size of the Omega subzone is also significantly decreased from eight residues to five. However, the structure of the cross-sheet ladder in this protein is similar to that of all other LexA endopeptidases.

### 2.8. Conformation of SCC During Self-Cleavage of LexA Endopeptidase Domain-like Family Proteins

With the lack of better X-ray LexA-like complexes with the RecA filament, several protein entries with PDB IDs: 8GMU, 8S7G, 8GMS, 8TRG, and 8GMT were chosen for the study, obtained by the electron microscopy method with a resolution (R) usually greater than 3 Å (see Section 2.1). Therefore, before accepting these structures, a structural comparison was made between the NucBaseOmega and Omega subzone areas of these structures and the X-ray template (Tables S5 and S6). The resulting data indicated that all the respective geometric parameters between the structures determined by two different methods numerically coincided well with each other. Minor differences were associated with the absence of the water molecule mediators (Table S5, column III) and some mutations of the catalytic dyad residues (Table S6, columns X and XI) that did not affect the conformation of the subzones. Thus, the several chosen electron microscopy structures in the areas of interest were deemed suitable to be used in the current study.

**Figure 5:**
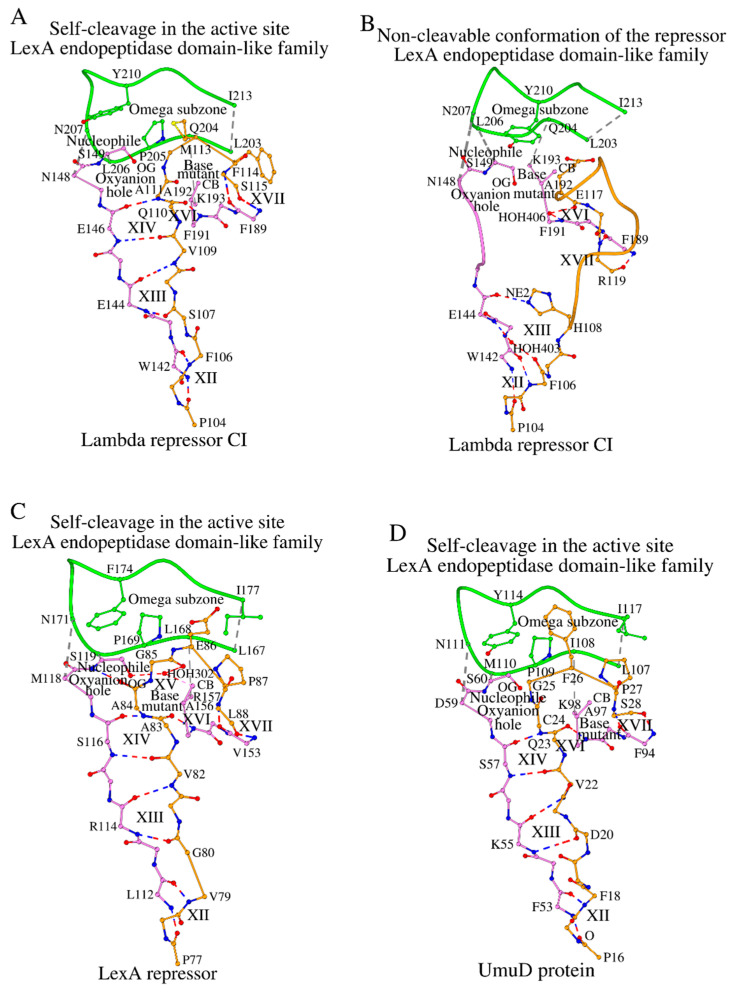
Four different types of interactions between proteins of the LexA endopeptidase domain-like family proteins with the target polypeptide chains. (**A**,**B**) show self-cleaved and non-cleavable variants of the lambda repressor CI, respectively, (**C**) shows LexA repressor, and (**D**) shows the UmuD protein. Interactions marked as XII through XVIII are described in Table S4.

The self-cleavage of the lambda repressor CI requires protein RecA in vivo. The 3D structure of lambda repressor CI in complex with a segment of RecA has been determined by electron microscopy (PDB ID: 8GMU, R = 2.78 Å) [19]. In 8GMU, the lambda repressor CI/RecA complex shows the catalytic domain of lambda repressor CI (Figure 5A). RecA affects the self-cleavage of lambda repressor CI at the Ala111-Gly112 peptide bond. Figure 5A shows the self-cleavage segment, Pro104-Ser115, bound to the rest of the protein. To prevent self-cleavage, the catalytic base, Lys192, was mutated to Ala192. The conformation of SCC during the self-cleavage of LexA endopeptidase domain-like proteins (Figure 5A) is similar to that of type I signal peptidases (Figure 4B), but there are clear differences. Before the point of protein cleavage, fragments Gly204-Pro212 (in signal peptidase IB) and Pro104-Gly112 (in lambda repressor CI) interact with the respective Nuc-peptides in an identical manner. However, after the point of protein cleavage, the conformation differs, where amino acids of the signal peptidase IB do cross-contact with the Omega subzone, while structurally similar amino acids of lambda repressor CI do not (Figure 4B vs. Figure 5A). In lambda repressor CI, the polypeptide chain turns around and the DAD_BASE_ (Phe189-Phe191 in lambda repressor CI) interactions XVI and XVII exist, but bind to substrate amino acids before (interaction XVI) and after (interaction XVII) the point of self-cleavage, unlike in signal peptidase IB, where the DAD_BASE_ motif is entirely bound to the substrate peptide prior to the point of peptide cleavage (Table S4).

### 2.9. Conformation of SCC in the Non-Activated (Unable to Self-Cleave) Form of Lambda Repressor CI

For the lambda repressor CI, there are also two sets of coordinates: RecA-activated 3D structures (PDB ID: 8GMU) [19] and the non-activated structures (PDB ID: 2HNF) [25] (Figure 5A and Figure 5B, respectively). The non-activated protein pattern does not show the typical parallel contact of the Nuc-peptide with the segment to be cleaved (Figure 5B vs. Figure 5A). Interactions structurally equivalent to XII and XIII do exist, and are followed by interactions XVI and XVII of the DAD_BASE_ motif (N/Phe189-O/Phe189-N/Phe191) with the tripeptide Glu117-Arg119 (Table S4). However, after activation by protein RecA, the conformation of the lambda repressor CI changes into the standard conformation (Figure 5A, Table S4).

### 2.10. Conformation of SCC During Self-Cleavage of LexA Repressors and UmuD Protein

With the same conformation of the SCC as above, we see in two LexA repressors the LexA repressor from *P. aeruginosa* (LEXA_PSEAI, PDB ID: 8S7G) [20] and the LexA repressor from *E. coli* (LEXA_ECOLI), in which there are three sets of coordinates with PDB IDs: 1JHE [21], 8GMS [19] and 8TRG [22] (Figure 5C, Table S4). A similar result is obtained for UmuD protein 3D structure (PDB ID: 8GMT [19] (Figure 5D).

### 2.11. SCC of the Standalone Protein 186 Repressor CI

**Figure 6:**
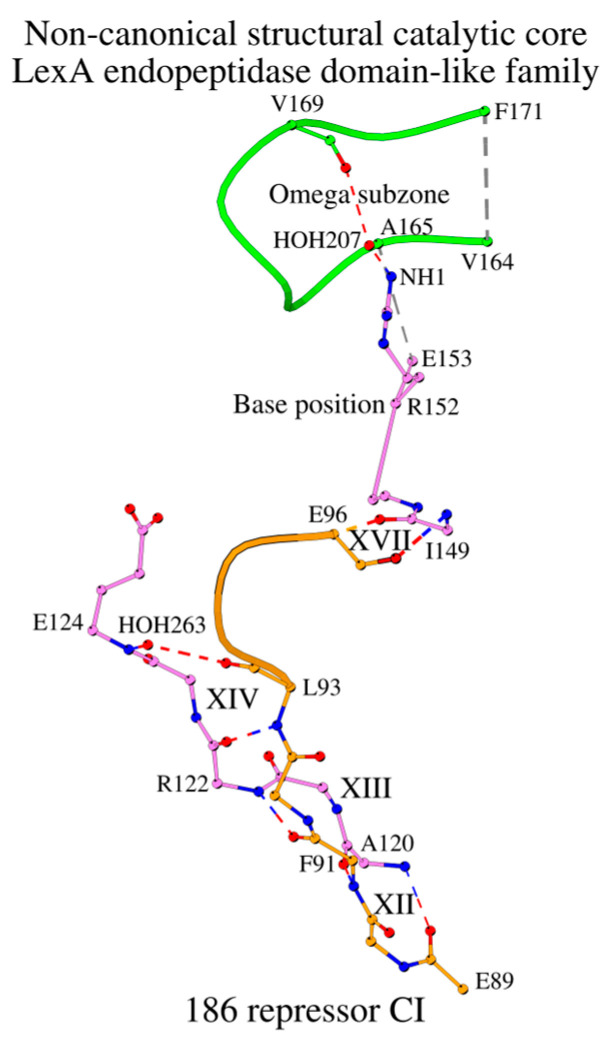
In the absence of a canonical active site due to the lack of both the C-terminal end of the Nuc-peptide and the catalytic base, the non-canonical SCC of the LexA endopeptidase domain-like protein 186 repressor CI interacts with the target polypeptide (shown orange color) in a unique way.

Section 2.7 mentioned the non-canonical SCC of the 186 repressor CI. However, this protein has a canonical cross-sheet ladder that begins with a tripeptide Met119-Ala120-Ile121 (Table 1 and Tables S1 and S2). Moreover, similarly to the complexes described here, fragment Ala120-Glu124 sandwiches the pentapeptide Glu89-Leu93 by means of interactions structurally equivalent to XII, XIII and XIV (Figure 6; columns XII-XIV in Table S4). Due to the absence of the C-terminal half of the Nuc-peptide, the next interaction in the SCC is only XVII, where Glu96 (equivalent to Ser115 in lambda repressor CI) contacts with the N-terminal part of the DAD_BASE_ motif (Figure 6; column XVII in Table S4). In an unrelated manner, but similarly to SipA (Figure 4C) which does not have peptidase activity, in 186 repressor CI there is an interaction between the amino acid at the position of the catalytic base (Arg152 in 186 repressor CI and Lys83 in SipA) and the main-chain oxygen atom (O/Val169 in 186 Repressor CI and O/Leu147 in SipA) of the Omega subzone. It is assumed that the molecular function of this protein is the lysogenic regulation in bacteriophage 186 [29].

### 2.12. The LexA/Signal Peptidase-like Superfamily: Existence of Two Protein Families with Different Molecular Function Correlates with Differences in Their Omega Subzones

The different direction of peptides after the point of cleavage in the families of type I signal peptidases and LexA endopeptidases can be explained by differences in the structure of their Omega subzones. These differences include: (1) the different size of the Omega subzone; (2) the appearance of a conservative proline (Pro205 in lambda repressor CI) at a specific place of the N-terminal segment of the Omega subzone, and of an aromatic residue (Tyr210 in lambda repressor CI) at the C-terminal segment of the Omega subzone in LexA endopeptidases; and (3) the existence of a residue with a large side-chain group in the second amino acid position after the cleavage point in the same family (Met113 in lambda repressor CI). Interactions of the DAD_BASE_ motif support the change in the polypeptide chain’s direction due to these differences.

### 2.13. Participation of the Cross-Sheet Ladder Step in Peptidase Dimerization

In the above study there were two structures of dimers without bound inhibitors, where the dimeric forms structurally demonstrated how a possible inter-chain function, such as self-cleavage or the inter-chain inhibition of catalytic activity, could take place. These structures are protein SipA from the family of type I signal peptidases (R9TES9_STRPY; PDB ID: 4N31; Figure 4C) and protein UmuD from the family of LexA endopeptidases (UMUD_ECOLI; PDB ID: 8GMT; Figure 5D).

**Figure 7:**
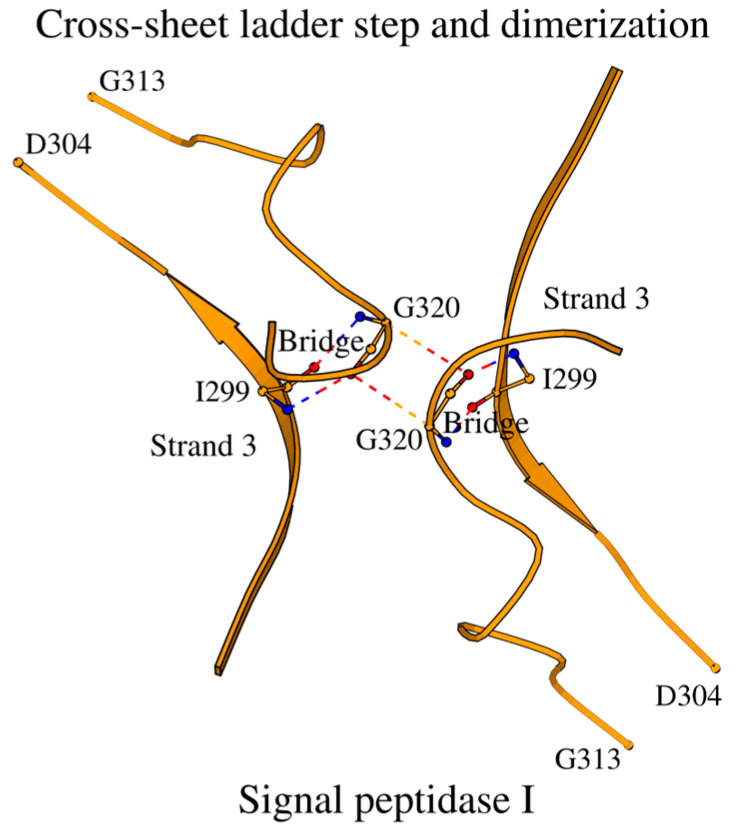
Dimer interface at step 3 of the cross-sheet ladder in type I signal peptidases.

Unlike these two structures, the signal dimer of the peptidase I from *E. coli* (LEP_ECOLI; PDB ID: 1B12; chains A and D) has a separate inhibitor in its active site. In LEP_ECOLI, the interface between chains A and D contains interacting glycine residues, which are located at position 320 at the C-terminus of the molecule: CA/Gly320_A/D-O/Gly320_D/A = 3.6/3.6 (2.6/2.7) 149°/140° (Figure 7). At the same time, Gly320 forms a bridge with the Ile299 of β-strand 3 of the same chain (see column “Dimer interface” in Table S3). In the structure with PDB ID: 1B12, the structural coordinates for the Lys305-Thr312 fragment are missing but can be found in the structure with PDB ID: 3IIQ of the same protein [33]. With this fragment, the dimer interface is also involved in Nuc-peptide stabilization and ligand binding with interactions Glu307-Pro87 and OE2/Glu307-O14/JZA314, respectively. Finally, Ile299, which forms a bridge with the interface Gly320, lies next to the cross-sheet ladder step Gly294-Ala298. Ten out of twelve representative structures show an identical mode of dimer formation (Table S3).

### 2.14. Comparison of the NucBaseOmega Subzones Between the LexA/Signal Peptidase-like Superfamily and the β-Lactamase/Transpeptidase-Like Superfamily

**Figure 8:**
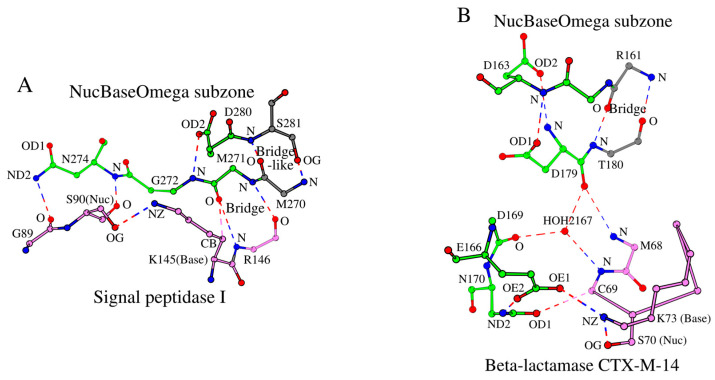
Comparison of the NucBaseOmega subzones in two different superfamilies of peptidases. (**A**) The LexA/signal peptidase-like superfamily with the example of signal peptidase I. (**B**) The β-lactamase/transpeptidase-like superfamily with the example of β-lactamase CTX-M-14.

Recently, a similarly derived SCC was introduced for the β-lactamase/transpeptidase-like superfamily [1]. As mentioned in the Introduction, there is a certain similarity in “function via a Ser-Lys catalytic dyad” between the β-lactamase/transpeptidases described in [1] and the LexA/signal peptidases described here. It is curious to see how their functional similarity translates into structural similarity between their SCCs. In addition to the catalytic residues and their respective subzones, the formation of the Omega subzone affects SCCs in the two superfamilies. Figure 8A and Figure 8B illustrate the NucBaseOmega subzones of the two superfamilies, using the examples of the signal peptidase I and the β-lactamase CTX-M-14, respectively.

The NucBaseOmega subzones of the two superfamilies differ significantly from each other, and they also differ in their folds, making the two superfamilies different locally and globally. Among similarities, one can generally pinpoint the presence of Asp, Asn and Glu residues in the region for both superfamilies. In particular, in both superfamilies, an asparagine of the Omega subzone, Asn274 and Asn170 in Figure 8A,B, interacts with the catalytic nucleophile and the residue preceding it.

## 3. Materials and Methods

The Protein Data Bank (PDB, http://www.rcsb.org/; 12 March 2025 [12]), Classification of protein families InterPro (https://www.ebi.ac.uk/interpro/entry/InterPro/IPR036286/; 12 March 2025 [3]) and the Structural Classification of Proteins database (SCOP, https://www.ebi.ac.uk/pdbe/scop/; 12 March 2025 [11]) were used to retrieve 19 representative structures of proteins from the LexA/signal peptidase-like superfamily (SCOP ID: 3000103). The probable quaternary structures were obtained using the PDBePISA server (https://www.ebi.ac.uk/pdbe/pisa/; 12 March 2025 [34]). Detailed structural information from the above set of PDB files is given in Section 2.1.

Structural visualization and the structural analysis of interactions between the amino acids in proteins (hydrogen bonds, hydrophobic, and other types of weak interactions) were conducted using Maestro (Schrödinger Release 2023-1: Schrödinger, LLC, New York, NY, USA, 2021; http://www.schrodinger.com/; 12 March 2025) and software [35] to determine interatomic contacts, i.e., of ligand–protein contacts (LPCs) and contacts of structural units (CSUs).

Weak hydrogen bonds were identified based on geometrical criteria [36]. Figures were drawn with MOLSCRIPT [37] and PyMOL molecular graphics system (version 3.1, https://pymol.org/; 12 March 2025).

## 4. Conclusions

Structural studies of the catalytic site of 12 representative proteins that are part of the LexA/signal peptidase-like superfamily have revealed that the B-type structural catalytic cores (B-SCCs) of these proteins consist of a combination of two conserved subzones: the NucBaseOmega subzone and the Omega subzone. The NucBaseOmega subzone incorporates (a) the Nuc-peptide, which is a hexapeptide, ending with the catalytic nucleophile; (b) the cross-sheet ladder, which is a set of bridge-like pair interactions, called steps, across the central β-sheet, and ending with the catalytic base; and (c) the N-terminal elements of the Omega subzone. The Omega subzone is a standalone, conserved substructure, which exists in two variants: (1) the Omega subzone of the type I signal peptidases, and (2) the Omega subzone of LexA endopeptidases. The latter variant is shorter than the prior, but a conservative proline and aromatic residue pair appear in it, which leads to a noticeable change in the local geometry between the two variants of Omega subzone.

Structural studies of the ligand binding and the self-cleavage process showed that proteins of the two families of the LexA/signal peptidase-like superfamily form complexes of two different types depending on the direction of the target polypeptide chain, which in turn is determined by differences in the structure of the C-terminal part of Omega subzone. The Nuc-peptide sandwiches the cleaved peptide in all enzymes such that the cleavage site is “correctly” placed near the catalytic nucleophile and the oxyanion hole. The catalytic base peptide (Xaa_BASE-3_-Xaa_BASE-2_-Xaa_BASE-1_-Lys_BASE_-Xaa_BASE+1_) forms a donor–acceptor–donor DAD_BASE_ motif (N/Xaa_BASE-3_-O/Xaa_BASE-3_-N/Xaa_BASE-1_) from the other side of the Nuc-peptide/substrate sandwich prior and after the point of cleavage. The conserved asparagine of the Omega subzone and the main-chain oxygen of its N-terminal residue interact with the catalytic nucleophile and catalytic base in all structures.

Finally, the cross-sheet ladder is a key scaffold for the SCC supporting the Nuc-peptide (at strand β1), the dimerization interface (at strand β3), the catalytic base (at strand β5) and the Omega subzone (at strand β6).

## Data Availability

The original contributions presented in this study are included in the article/Supplementary Material. Further inquiries can be directed to the corresponding authors.

## Supplementary Materials

The supporting information can be downloaded at https://www.mdpi.com/article/10.3390/ijms27052127/s1.
