# Supplementary material for: The Role of the Omega Subzone in Determining the Membership of a Protein in One of the Two Families of the LexA/Signal Peptidase-like Superfamily
Source: Int J Mol Sci. 2026 Feb 25;27(5):2127. doi: 10.3390/ijms27052127 (PMC12984324; doi:10.3390/ijms27052127)
Supplement: Supplementary file 1 [file ijms-27-02127-s001.zip › ijms-4146843-supplementary.pdf]

Supplementary Materials

The Role of the Omega Subzone in Determining Protein's Membership to One of the Two Families of the LexA/Signal Peptidase-Like Superfamily

Alexander I. Denesyuk [1], Konstantin Denessiouk [1], Mark S. Johnson [1] and Vladimir N. Uversky [2]

[1] Structural Bioinformatics Laboratory, Biochemistry, InFLAMES Research Flagship Center, Faculty of Science and Engineering, Åbo Akademi University, Turku 20520, Finland

[2] Department of Molecular Medicine and USF Health Byrd Alzheimer's Research Institute, Morsani College of Medicine, University of South Florida, Tampa, FL 33612, USA

Table S1. Two patterns of the cross-sheet ladders in 12 LexA/signal peptidase-like superfamily representative proteins.

N	PDB ID	Protein	Nuc octa/hexapeptide	Omega subzone	Cross-sheet ladder
			Superfamily: LexA/signal peptidase-like		
			Family: Type I signal peptidase		
1a	1B12_A	LEP_ECOLI	83 PFQIPSG**S** 90	272 GDNRDNSAD 280	E 82; 103 VE 104;294 GR 295;132 VF 133; Y 143
1b	≡	≡	85 --QIPSG**S** 90	≡	F 84; 101 IL 102;296ATA 298;130 IV 131;145 **K**R 146;270 MM 271; S 281
2a	4WVI_A	LEP_STAAC	380 PYTIKGE**S** 387	497 GDNREVSKD 505	T 379;400 VN 401;519 GK 520;417 VF 418; Y 426
2b	≡	≡	382 --TIKGE**S** 387	≡	Y 381;398 VA 399;521VS- 522;415 VV 416;428 **K**R 429;495 VL 496; S 506
3a	4N31_A	R9TES9_STRPY	41 VMIINTND 48	140 NDYREERLD 148	G 40; 61 YY 62; 162 GK 163; 75 VY 76; K 83
3b	≡	≡	43 --IINTND 48	≡	M 42; 59 VL 60; 164IST 166; 73 VV 74; 85 GR 86; 138 IL 139; S 149
4a	7P2P_A	SC11A_HUMAN	49 IVVVLSG**S** 56	115 GDNNA--VD 121	P 48; 69 LT 70; 140 GR 141; 84 VF 85; I 94
4b	≡	≡	51 --VVLSG**S** 56	≡	V 50; 67 LF 68; 142ARG 144; 82 IV 83; 96 **H**R 97; 113 TK 114; D 122
			Family: LexA endopeptidase domain-like		
5a	1F39_A	RPC1_LAMBD	142 WLEVEGN**S** 149	205 PLNPQ-YPM 212	F 141;168 VD 169;223 GK 224;181 IA 182; T 190
5b	≡	≡	144 --EVEGN**S** 149	≡	L 143;166 IL 167;225VIA 227;179 FC 180;192 **K**K 193;203 LQ 204; I 213
6a	2HNF_A	Q7B004_ECOLX	142 WLEVEGN**S** 149	205 PLNPQ-YPM 212	F 141;168 VD 169;223 GK 224;181 IA 182; T 190
6b	≡	≡	144 --EVEGN**S** 149	≡	L 143;166 IL 167;225VIA 227;179 FC 180;192 AK 193;203 LQ 204; I 213
7a	3K2Z_A	LEXA_THEMA	112 LLKVKGE**S** 119	169 PANRE-MSS 176	F 111;133 VR 134;188 GK 189;146 AA 147; T 154
7b	≡	≡	114 --KVKGE**S** 119	≡	L 113;131 VL 132;190VVG 192;144 IV 145;156 AK 157;167 LR 168; M 177
8a	6A2Q_A	LEXA_MYCTU	153 LLKVIGD**S** 160	210 PHNPA-FDP 217	F 152;174 VR 175;227 GK 228;187 AA 188; T 195
8b	≡	≡	155 --KVIGD**S** 160	≡	L 154;172 VV 173;229VVT 231;185 IV 186;197 **K**T 198;208 LM 209; I 218
9a	8B0V_A	LEXA_PSEAI	118 LLRVRGM**S** 125	175 AENPE-FAP 182	Y 117;139 VH 140;196 GL 197;152 VA 153; T 160
9b	≡	≡	120 --RVRGM**S** 125	≡	L 119;137 LA 138;198SVG 200;150 VV 151;162 **K**R 163;173 LL 174; I 183
10a	1JHF_A	LEXA_ECOLI	112 LLRVSGM**S** 119	169 PENSE-FKP 176	F 111;133 VH 134;190 GL 191;146 VA 147; T 154
10b	≡	≡	114 --RVSGM**S** 119	≡	L 113;131 LA 132;192AVG 194;144 VV 145;156 **K**R 157;167 LL 168; I 177
11a	1UMU_A	UMUD_ECOLI	53 FVKASGD**S** 60	109 PMNSA-YSP 116	Y 52; 74 VD 75; 129 GV 130; 87 IA 88; T 95
11b	≡	≡	55 --KASGD**S** 60	≡	V 54; 72 LI 73; 131VIH 133; 85 IV 86; 97 **K**K 98; 107 LI 108; I 117
12a	2FJR_A	RPC1_BP186	120 AIRS---- 123	166 GG----KVP 170	M 119;130 VD 131;182 GR 183;142 LV 143; S 150
12b	≡	≡	122 --RS---- 123	≡	I 121;128 YF 129;184VVG 186;140 LW 141;152 RE 153;164 VA 165; F 171

Table S2. Conserved geometric parameters (distance and angle) in two patterns of the cross-sheet ladder contacts in 12 LexA/signal peptidase-like superfamily representative proteins.

N	PDB ID	Protein	I	II	III	IV	V	VI
				Superfamily: LexA/signal peptidase-like				
				Family: Type I signal peptidase				
1a	1B12_A	LEP_ECOLI	N/E$_{82}$-O/V$_{103}$ 2.9	N/E$_{104}$-O/R$_{295}$ 2.8	N/G$_{294}$-O/V$_{132}$ 3.2	N/F$_{133}$-O/Y$_{143}$ 3.0	N/A	N/A
			O/E$_{82}$-N/V$_{103}$ 2.8	O/E$_{104}$-N/R$_{295}$ 3.0	O/G$_{294}$-N/V$_{132}$ 2.8	O/F$_{133}$-N/Y$_{143}$ 2.8		
1b			N/F$_{84}$-O/I$_{101}$ 2.8	N/L$_{102}$-O/A$_{298}$ 2.9	N/A$_{296}$-O/I$_{130}$ 3.0	N/V$_{131}$-O/K$_{145}$ 2.7	N/R$_{146}$-O/M$_{271}$ 3.0	N/M$_{270}$-OG/S$_{281}$ 2.8
	≡	≡	O/F$_{84}$-N/I$_{101}$ 2.9	O/L$_{102}$-N/T$_{297}$ 2.8	O/A$_{296}$-HOH$_{1034}$ 3.1	O/V$_{131}$-N/K$_{145}$ 3.0	O/R$_{146}$-N/M$_{271}$ 3.0	O/M$_{270}$-N/S$_{281}$ 2.9
					HOH$_{1034}$-N/I$_{130}$ 2.8			
2a	4WVI_A	LEP_STAAC	N/T$_{379}$-O/V$_{400}$ 2.9	N/N$_{401}$-O/K$_{520}$ 2.7	N/G$_{519}$-O/V$_{417}$ 3.4	N/F$_{418}$-O/Y$_{426}$ 2.9	N/A	N/A
			O/T$_{379}$-N/V$_{400}$ 2.8	O/N$_{401}$-N/K$_{520}$ 2.9	O/G$_{519}$-N/V$_{417}$ 2.9	O/F$_{418}$-N/Y$_{426}$ 2.8		
2b			N/Y$_{381}$-O/V$_{398}$ 2.9	N/A$_{399}$-HOH$_{814}$ 2.9	N/V$_{521}$-O/V$_{415}$ 2.8	N/V$_{416}$-O/K$_{428}$ 2.8	N/R$_{429}$-O/L$_{496}$ 2.8	N/V$_{495}$-OG/S$_{506}$ 2.9
	≡	≡	O/Y$_{381}$-N/V$_{398}$ 2.8	HOH$_{814}$-O/S$_{522}$ 2.5	O/V$_{521}$-HOH$_{810}$ 2.8	O/V$_{416}$-N/K$_{428}$ 3.1	O/R$_{429}$-N/L$_{496}$ 2.9	O/V$_{495}$-N/S$_{506}$ 2.9
				O/A$_{399}$-N/S$_{522}$ 2.9	HOH$_{810}$-N/V$_{415}$ 2.9			
3a	4N31_A	R9TES9_STRPY	N/G$_{40}$-O/Y$_{61}$ 2.9	N/Y$_{62}$-O/K$_{163}$ 3.0	N/G$_{162}$-O/V$_{75}$ 3.5	N/Y$_{76}$-O/K$_{83}$ 2.8	N/A	N/A
			O/G$_{40}$-N/Y$_{61}$ 2.9	O/Y$_{62}$-N/K$_{163}$ 2.9	O/G$_{162}$-N/V$_{75}$ 3.0	O/Y$_{76}$-N/K$_{83}$ 2.7		
3b			N/M$_{42}$-O/V$_{59}$ 2.9	N/L$_{60}$-O/T$_{166}$ 3.0	N/I$_{164}$-O/V$_{73}$ 2.8	N/V$_{74}$-O/G$_{85}$ 2.8	N/R$_{86}$-O/L$_{139}$ 3.1	N/I$_{138}$-OG/S$_{149}$ 2.9
	≡	≡	O/M$_{42}$-N/V$_{59}$ 2.8	O/L$_{60}$-N/S$_{165}$ 2.9	O/I$_{164}$-HOH$_{305}$ 2.7	O/V$_{74}$-N/G$_{85}$ 3.0	O/R$_{86}$-N/L$_{139}$ 2.9	O/I$_{138}$-N/S$_{149}$ 2.8
					HOH$_{305}$-N/V$_{73}$ 2.9			
4a	7P2P_A	SC11A_HUMAN	N/A	N/T$_{70}$-O/R$_{141}$ 2.4	N/G$_{140}$-O/V$_{84}$ 3.8	N/F$_{85}$-O/I$_{94}$ 2.9	N/A	N/A
			O/P$_{48}$-CB/L$_{69}$	O/T$_{70}$-N/R$_{141}$ 2.8	O/G$_{140}$-N/V$_{84}$ 3.8	O/F$_{85}$-N/I$_{94}$ 2.9		
			4.3 (3.5) 138°					
4b	≡	≡	N/V$_{50}$-O/L$_{67}$ 3.0	N/F$_{68}$-O/G$_{144}$ 2.9	N/A$_{142}$-O/I$_{82}$ 5.0	N/V$_{83}$-O/H$_{96}$ 2.9	N/R$_{97}$-O/K$_{114}$ 4.1	OG1/T$_{113}$-OD2/D$_{122}$ 3.2
			O/V$_{50}$-N/L$_{67}$ 2.8	O/F$_{68}$-N/R$_{143}$ 4.5	O/A$_{142}$-N/I$_{82}$ 6.6	O/V$_{83}$-N/H$_{96}$ 2.9	O/R$_{97}$-N/K$_{114}$ 3.4	O/T$_{113}$-N/D$_{122}$ 3.9
				Family: LexA endopeptidase domain-like				
5a	1F39_A	RPC1_LAMBD	N/F$_{141}$-O/V$_{168}$ 2.8	N/D$_{169}$-O/K$_{224}$ 2.8	N/G$_{223}$-O/I$_{181}$ 3.4	N/A$_{182}$-O/T$_{190}$ 2.8	N/A	N/A
			O/F$_{141}$-N/V$_{168}$ 2.8	O/D$_{169}$-N/K$_{224}$ 3.1	O/G$_{223}$-N/I$_{181}$ 3.0	O/A$_{182}$-N/T$_{190}$ 3.0		
5b			N/L$_{143}$-O/I$_{166}$ 2.9	N/L$_{167}$-O/A$_{227}$ 2.8	N/V$_{225}$-O/F$_{179}$ 3.0	N/C$_{180}$-O/K$_{192}$ 2.9	N/K$_{193}$-O/Q$_{204}$ 2.8	N/L$_{203}$-O/I$_{213}$ 2.7
	≡	≡	O/L$_{143}$-N/I$_{166}$ 2.9	O/L$_{167}$-N/I$_{226}$ 2.8	O/V$_{225}$-HOH$_{31}$ 2.9	O/C$_{180}$-N/K$_{192}$ 3.1	O/K$_{193}$N/Q$_{204}$ 2.7	O/L$_{203}$-N/I$_{213}$ 2.9
					HOH$_{31}$-N/F$_{179}$ 2.8			
6a	2HNF_A	Q7B004_ECOLX	N/F$_{141}$-O/V$_{168}$ 3.0	N/D$_{169}$-O/K$_{224}$ 2.8	N/G$_{223}$-O/I$_{181}$ 3.4	N/A$_{182}$-O/T$_{190}$ 3.0	N/A	N/A
			O/F$_{141}$-N/V$_{168}$ 2.7	O/D$_{169}$-N/K$_{224}$ 3.1	O/G$_{223}$-N/I$_{181}$ 3.1	O/A$_{182}$-N/T$_{190}$ 2.9		
6b			N/L$_{143}$-O/I$_{166}$ 3.0	N/L$_{167}$-O/A$_{227}$ 2.8	N/V$_{225}$-O/F$_{179}$ 3.0	N/C$_{180}$-O/A$_{192}$ 3.0	N/K$_{193}$-O/Q$_{204}$ 3.1	N/L$_{203}$-O/I$_{213}$ 2.7
	≡	≡	O/L$_{143}$-N/I$_{166}$ 2.9	O/L$_{167}$-N/I$_{226}$ 2.9	O/V$_{225}$-HOH$_{415}$ 2.8	O/C$_{180}$-N/A$_{192}$ 3.2	O/K$_{193}$N/Q$_{204}$ 2.8	O/L$_{203}$-N/I$_{213}$ 2.9
					HOH$_{415}$-N/F$_{179}$ 2.9			
	1UMU_A	UMUD_ECOLI	N/Y$_{52}$-O/V$_{74}$ 2.9	N/D$_{75}$-O/V$_{130}$ 3.0	N/G$_{129}$-O/I$_{87}$ 2.8	N/A$_{88}$-O/T$_{95}$ 2.8	N/A	N/A
			O/Y$_{52}$-N/V$_{74}$ 2.7	O/D$_{75}$-N/V$_{130}$ 3.1	O/G$_{129}$-N/I$_{87}$ 3.2	O/A$_{88}$-N/T$_{95}$ 2.7		
			N/V$_{54}$-O/L$_{72}$ 3.0	N/I$_{73}$-O/H$_{133}$ 2.9	N/V$_{131}$-O/I$_{85}$ 2.8	N/V$_{86}$-O/K$_{97}$ 2.8	N/K$_{98}$-O/I$_{108}$ 3.1	N/L$_{107}$-O/I$_{117}$ 2.7
	≡	≡	O/V$_{54}$-N/L$_{72}$ 2.7	O/I$_{73}$-N/I$_{132}$ 2.8	O/V$_{131}$-HOH$_{141}$ 2.8	O/V$_{86}$-N/K$_{97}$ 3.0	O/K$_{98}$-N/I$_{108}$ 3.0	O/L$_{107}$-N/I$_{117}$ 2.9
					HOH$_{141}$-N/I$_{85}$ 2.8			

7a	3K2Z_A	LEXA_THEMA	N/F$_{111}$-O/V$_{133}$ 2.9	N/R$_{134}$-O/K$_{189}$ 2.9	N/G$_{188}$-O/A$_{146}$ 3.4	N/A$_{147}$-O/T$_{154}$ 3.0	N/A	N/A
			O/F$_{111}$-N/V$_{133}$ 2.7	O/R$_{134}$-N/K$_{189}$ 2.9	O/G$_{188}$-N/A$_{146}$ 3.0	O/A$_{147}$-N/T$_{154}$ 2.9		
7b			N/L$_{113}$-O/V$_{131}$ 3.0	N/L$_{132}$-O/G$_{192}$ 2.9	N/V$_{190}$-O/I$_{144}$ 2.8	N/V$_{145}$-O/A$_{156}$ 2.8	N/K$_{157}$-O/R$_{168}$ 2.8	N/L$_{167}$-O/M$_{177}$ 2.8
	≡	≡	O/L$_{113}$-N/V$_{131}$ 2.9	O/L$_{132}$-N/V$_{191}$ 2.8	O/V$_{190}$-HOH$_{585}$ 2.8<br>HOH$_{585}$-N/I$_{144}$ 2.9	O/V$_{145}$-N/A$_{156}$ 3.2	O/K$_{157}$-N/R$_{168}$ 2.9	O/L$_{167}$-N/M$_{177}$ 3.2
8a	6A2Q_A	LEXA_MYCTU	N/F$_{152}$-O/V$_{174}$ 2.9	N/R$_{175}$-O/K$_{228}$ 2.8	N/G$_{227}$-O/A$_{187}$ 3.4	N/A$_{188}$-O/T$_{195}$ 2.9	N/A	N/A
			O/F$_{152}$-N/V$_{174}$ 2.7	O/R$_{175}$-N/K$_{228}$ 2.9	O/G$_{227}$-N/A$_{187}$ 2.9	O/A$_{188}$-N/T$_{195}$ 2.8		
8b			N/L$_{154}$-O/V$_{172}$ 2.9	N/V$_{173}$-O/T$_{231}$ 2.9	N/V$_{229}$-O/I$_{185}$ 2.8	N/V$_{186}$-O/K$_{197}$ 2.8	N/T$_{198}$-O/M$_{209}$ 2.9	N/L$_{208}$-O/I$_{218}$ 2.8
	≡	≡	O/L$_{154}$-N/V$_{172}$ 2.8	O/V$_{173}$-N/V$_{230}$ 2.9	O/V$_{229}$-HOH$_{470}$ 2.8<br>HOH$_{470}$-N/I$_{185}$ 3.0	O/V$_{186}$-N/K$_{197}$ 3.0	O/T$_{198}$-N/M$_{209}$ 2.7	O/L$_{208}$-N/I$_{218}$ 2.9
9a	8B0V_A	LEXA_PSEAI	N/Y$_{117}$-O/V$_{139}$ 3.4	N/H$_{140}$-O/L$_{197}$ 2.9	N/E$_{195}$-O/V$_{152}$ 2.8	N/A$_{153}$-O/T$_{160}$ 2.9	N/A	N/A
			O/Y$_{117}$-N/V$_{139}$ 2.9	O/H$_{140}$-N/L$_{197}$ 2.9	O/G$_{196}$-N/V$_{152}$ 2.9	O/A$_{153}$-N/T$_{160}$ 2.9		
9b	≡	≡	N/L$_{119}$-O/L$_{137}$ 2.8	N/A$_{138}$-O/G$_{200}$ 2.9	N/S$_{198}$-O/V$_{150}$ 2.9	N/V$_{151}$-O/K$_{162}$ 2.9	N/R$_{163}$-O/L$_{174}$ 2.9	N/L$_{173}$-O/I$_{183}$ 2.7
			O/L$_{119}$-N/L$_{137}$ 2.8	O/A$_{138}$-N/V$_{199}$ 2.8	O/S$_{198}$-HOH$_{415}$ 4.9<br>HOH$_{415}$-N/V$_{150}$ 3.0	O/V$_{151}$-N/K$_{162}$ 3.1	O/R$_{163}$-N/L$_{174}$ 2.8	O/L$_{173}$-N/I$_{183}$ 3.0
10a	1JHF_A	LEXA_ECOLI	N/F$_{111}$-O/V$_{133}$ 3.5	N/H$_{134}$-O/L$_{191}$ 2.9	N/G$_{190}$-O/V$_{146}$ 3.4	N/A$_{147}$-O/T$_{154}$ 3.0	N/A	N/A
			O/F$_{111}$-N/V$_{133}$ 2.9	O/H$_{134}$-N/L$_{191}$ 3.0	O/G$_{190}$-N/V$_{146}$ 3.0	O/A$_{147}$-N/T$_{154}$ 2.9		
10b			N/L$_{113}$-O/L$_{131}$ 2.9	N/A$_{132}$-O/G$_{194}$ 2.9	N/A$_{192}$-O/V$_{144}$ 3.2	N/V$_{145}$-O/K$_{156}$ 2.9	N/R$_{157}$-O/L$_{168}$ 3.0	N/L$_{167}$-O/I$_{177}$ 2.9
	≡	≡	O/L$_{113}$-N/L$_{131}$ 2.9	O/A$_{132}$-N/V$_{193}$ 2.9	O/A$_{192}$-HOH$_{433}$ 2.7<br>HOH$_{433}$-N/V$_{144}$ 3.4	O/V$_{145}$-N/K$_{156}$ 3.2	O/R$_{157}$-N/L$_{168}$ 2.9	O/L$_{167}$-N/I$_{177}$ 3.1
11a	1UMU_A	UMUD_ECOLI	N/Y$_{52}$-O/V$_{74}$ 2.9	N/D$_{75}$-O/V$_{130}$ 3.0	N/G$_{129}$-O/I$_{87}$ 2.8	N/A$_{88}$-O/T$_{95}$ 2.8	N/A	N/A
			O/Y$_{52}$-N/V$_{74}$ 2.7	O/D$_{75}$-N/V$_{130}$ 3.1	O/G$_{129}$-N/I$_{87}$ 3.2	O/A$_{88}$-N/T$_{95}$ 2.7		
11b	≡	≡	N/V$_{54}$-O/L$_{72}$ 3.0	N/I$_{73}$-O/H$_{133}$ 2.9	N/V$_{131}$-O/I$_{85}$ 2.8	N/V$_{86}$-O/K$_{97}$ 2.8	N/K$_{98}$-O/I$_{108}$ 3.1	N/L$_{107}$-O/I$_{117}$ 2.7
			O/V$_{54}$-N/L$_{72}$ 2.7	O/I$_{73}$-N/I$_{132}$ 2.8	O/V$_{131}$-HOH$_{141}$ 2.8<br>HOH$_{141}$-N/I$_{85}$ 2.8	O/V$_{86}$-N/K$_{97}$ 3.0	O/K$_{98}$-N/I$_{108}$ 3.0	O/L$_{107}$-N/I$_{117}$ 2.9
12a	2FJR_A	RPC1_BP186	N/M$_{119}$-O/V$_{130}$ 3.0	N/D$_{131}$-O/R$_{183}$ 2.8	N/G$_{182}$-O/L$_{142}$ 3.4	N/V$_{143}$-O/S$_{150}$ 2.9	N/A	N/A
			O/M$_{119}$-N/V$_{130}$ 2.8	O/D$_{131}$-N/R$_{183}$ 3.0	O/G$_{182}$-N/L$_{142}$ 2.9	O/V$_{143}$-N/S$_{150}$ 3.1		
12b			N/I$_{121}$-O/Y$_{128}$ 2.8	N/F$_{129}$-O/G$_{186}$ 2.8	N/V$_{184}$-O/L$_{140}$ 2.8	N/W$_{141}$-O/R$_{152}$ 2.8	N/E$_{153}$-O/A$_{165}$ 2.8	N/V$_{164}$-O/F$_{171}$ 2.8
	≡	≡	O/I$_{121}$-N/Y$_{128}$ 2.7	O/F$_{129}$-N/V$_{185}$ 2.8	O/V$_{184}$-HOH$_{198}$ 2.9<br>HOH$_{198}$-N/L$_{140}$ 2.9	O/W$_{141}$-N/R$_{152}$ 2.8	O/E$_{153}$-N/A$_{165}$ 2.9	O/V$_{164}$-N/F$_{171}$ 3.0

N/A–Not Available.

Table S3. Conserved geometric parameters (distance and angle) of the Omega and NucBaseOmega subzones in 12 LexA/signal peptidase-like superfamily representative proteins.

N	PDB ID	Protein	VII	VIII	IX	X	XI	Dimer interface
				Superfamily: LexA/signal peptidase-like<br>Family: Type I signal peptidase				
1	1B12_A, D	LEP_ECOLI	N/G$_{272}$-OD2/D$_{280}$ 3.1	O/N$_{274}$-N/N$_{277}$ 2.9<br>OD1/N$_{274}$-ND2/N$_{277}$ 3.1	O/S$_{90}$-N/N$_{274}$ 3.0<br>O/G$_{89}$-ND2/N$_{274}$ 2.8	CB/K$_{145}$-O/M$_{271}$<br>3.7 (2.7) 156°	OG/S$_{90}$-NZ/K$_{145}$ 3.0	N/I$_{299_A/D}$-O/G$_{320_A/D}$ 2.9/2.9<br>O/I$_{299_A/D}$-N/G$_{320_A/D}$ 2.9/2.9<br>homodimer
2	4WVI_A	LEP_STAAC	N/G$_{497}$-OD2/D$_{505}$ 3.0	O/N$_{499}$-N/V$_{502}$ 3.0<br>OD1/N$_{499}$-CG2/V$_{502}$<br>4.0 (2.9) 160°	O/S$_{387}$-N/N$_{499}$ 3.0<br>O/E$_{386}$-ND2/N$_{499}$ 2.9	CB/K$_{428}$-O/L$_{496}$<br>3.7 (2.7) 149°	OG/S$_{387}$-HOH$_{857}$ 2.8<br>HOH$_{857}$-NZ/K$_{428}$ 3.4	N/A
3	4N31_A, B	R9TES9_STRPY	N/N$_{140}$-OD2/D$_{148}$ 2.8	O/Y$_{142}$-N/E$_{145}$ 3.0<br>CE2/Y$_{142}$-OE2/E$_{145}$<br>4.1 (3.1) 146°	O/D$_{48}$-N/Y$_{142}$ 3.0<br>O/N$_{47}$-CB/Y$_{142}$<br>3.6 (3.1) 108°	CA/G$_{85}$-O/L$_{139}$<br>4.0 (3.6) 100°	N/A	N/L$_{167_A/B}$-O/R$_{169_B/A}$ 2.8/2.9<br>O/L$_{167_A/B}$-N/R$_{169_B/A}$ 2.9/3.0<br>homodimer
4	7P2P_A, B	SC11A_HUMAN	N/G$_{115}$-OD1/D$_{122}$ 3.7	O/D$_{116}$-CB/S$_{56}$<br>4.3 (3.4) 133°<br>O/D$_{116}$-ND2/N$_{118}$ 3.2	O/S$_{56}$-ND2/N$_{117}$ 3.4<br>O/G$_{55}$-N/E$_{58}$ 3.8	CB/H$_{96}$-O/K$_{114}$<br>5.2 (4.1) 173°	OG/S$_{56}$-NE2/H$_{96}$ 5.8	N/F$_{145_A}$-O/G$_{155_B}$ 3.5<br>O/R$_{143_A}$-N/L$_{157_B}$ 3.0<br>heterodimer
				Family: LexA endopeptidase domain-like				
5	1F39_A, B	RPC1_LAMBD	CG/P$_{205}$-O/P$_{211}$<br>3.5 (2.7) 132°	O/N$_{207}$-N/Y$_{210}$ 2.9<br>OD1/N$_{207}$-N/Q$_{209}$ 3.0	O/S$_{149}$-N/N$_{207}$ 3.1<br>O/N$_{148}$-ND2/N$_{207}$ 2.9	CB/K$_{192}$-O/Q$_{204}$<br>3.7 (2.7) 142°	OG/S$_{149}$-NZ/K$_{192}$ 3.1	N/S$_{228_A/B}$-HOH$_{1/2}$ 2.9/3.0<br>HOH$_{1/2}$-O/W$_{230_B/A}$ 2.8/2.7<br>O/S$_{228_A/B}$-N/W$_{230_B/A}$ 3.0/3.0<br>homodimer
6	2HNF_A	Q7B004_ECOLX	CG/P$_{205}$-O/P$_{211}$<br>3.4 (2.8) 116°	O/N$_{207}$-N/Y$_{210}$ 2.9<br>OD1/N$_{207}$-N/Q$_{209}$ 2.9	O/S$_{149}$-N/N$_{207}$ 3.0<br>O/N$_{148}$-ND2/N$_{207}$ 3.0	CB/A$_{192}$-O/Q$_{204}$<br>3.8 (2.8) 147°	OG/S$_{149}$-HOH$_{413}$ 2.9<br>HOH$_{413}$-CB/A$_{192}$<br>4.6 (3.7) 148°	N/A
7	3K2Z_A, B	LEXA_THEMA	CG/P$_{169}$-O/S$_{175}$<br>3.7 (2.9) 131°	O/N$_{171}$-N/M$_{174}$ 3.0<br>OD1/N$_{171}$-N/E$_{173}$ 2.8	O/S$_{119}$-N/A$_{170}$ 2.7<br>O/E$_{118}$-ND2/N$_{171}$ 2.9	CB/A$_{156}$-O/R$_{168}$<br>3.7 (2.8) 132°	N/A	N/V$_{193_A/B}$-O/R$_{195_B/A}$ 2.9/2.9<br>O/V$_{193_A/B}$-N/R$_{195_B/A}$ 2.8/2.9<br>homodimer
8	6A2Q_A, C	LEXA_MYCTU	CG/P$_{210}$-O/D$_{216}$<br>3.7 (2.9) 135°	O/N$_{212}$-N/F$_{215}$ 3.0<br>OD1/N$_{212}$-N/A$_{214}$ 2.8	O/S$_{160}$-N/H$_{211}$ 2.8<br>O/D$_{159}$-ND2/N$_{212}$ 2.8	CB/K$_{197}$-O/M$_{209}$<br>3.7 (2.8) 131°	OG/S$_{160}$-NZ/K$_{197}$ 3.1	N/V$_{232_A/C}$-O/R$_{234_C/A}$ 2.8/2.8<br>O/V$_{232_A/C}$-N/R$_{234_C/A}$ 2.8/2.8<br>homodimer
9	8B0V_A<br>8S7G_A, B	LEXA_PSEAI<br>LEXA_PSEAI	CB/A$_{175}$-HOH$_{416}$<br>3.7 (2.8) 138°<br>HOH$_{416}$-O/A$_{181}$ 2.9	O/N$_{177}$-N/F$_{180}$ 3.0<br>OD1/N$_{177}$-N/E$_{179}$ 3.0	O/S$_{125}$-N/E$_{176}$ 2.8<br>O/M$_{124}$-ND2/N$_{177}$ 2.8	CB/K$_{162}$-O/L$_{174}$<br>3.6 (2.9) 124°	OG/S$_{125}$-NZ/K$_{162}$ 2.8	O/V$_{201_A}$-N/R$_{203_B}$ 3.2<br>N/V$_{201_B}$-O/R$_{203_A}$ 3.4<br>homodimer
10	1JHF_A, B	LEXA_ECOLI	CG/P$_{169}$-O/K$_{175}$<br>4.1 (3.2) 137°	O/N$_{171}$-N/F$_{174}$ 3.1<br>OD1/N$_{171}$-N/E$_{173}$ 2.9	O/S$_{119}$-N/E$_{170}$ 2.8<br>O/M$_{118}$-ND2/N$_{171}$ 2.9	CB/K$_{156}$-O/L$_{168}$<br>3.7 (3.0) 121°	OG/S$_{119}$-NZ/K$_{156}$ 2.9	N/V$_{195_A/B}$-O/R$_{197_B/A}$ 3.0/3.0<br>O/V$_{195_A/B}$-N/R$_{197_B/A}$ 3.0/2.9<br>homodimer
11	1UMU_A, B	UMUD_ECOLI	CG/P$_{109}$-O/S$_{115}$<br>4.2 (3.5) 127°	O/N$_{111}$-N/Y$_{114}$ 2.9<br>OD1/N$_{111}$-N/A$_{113}$ 2.8	O/S$_{60}$-N/M$_{110}$ 2.7<br>O/D$_{59}$-ND2/N$_{111}$ 2.7	CB/K$_{97}$-O/I$_{108}$<br>3.9 (3.0) 135°	OG/S$_{60}$-NZ/K$_{97}$ 2.8	N/V$_{134_A/B}$-O/K$_{136_B/A}$ 3.1/3.7<br>O/V$_{134_A/B}$-N/K$_{136_B/A}$ 2.7/2.8<br>homodimer
12	2FJR_A	RPC1_BP186	N/G$_{167}$-HOH$_{207}$ 2.8<br>HOH$_{207}$-O/V$_{169}$ 3.1	N/A	N/A	CB/R$_{152}$-<br>O/A$_{165}$<br>3.2 (2.4) 133°	N/A	O/V$_{187_A/B}$-N/S$_{189_B/A}$ 2.9/2.9<br>N/V$_{187_A/B}$-O/S$_{189_B/A}$ 2.9/3.0<br>homodimer

N/A–Not Available.

Table S4. Conserved geometric parameters (distance and angle) of interactions between SCC and an inhibitor or the target polypeptide chain in 9 LexA/signal peptidase-like superfamily representative proteins.

N	PDB ID	Protein	XII	XIII	XIV	Oxy and XV	XVI	XVII	XVIII
					Superfamily: LexA/signal peptidase-like				
					Family: Type I signal peptidase				
1	1B12_A	LEP_ECOLI	N/A	N/A<br>O/Q$_{85}$-HOH$_{1050}$ 2.6<br>HOH$_{1050}$-O19/1PN$_{1001}$ 3.7	CD/P$_{87}$-O19/1PN$_{1001}$ 3.9 (2.9) 158°<br>N/A	N/S$_{90}$-O8/1PN$_{1001}$ 3.0<br>NZ/K$_{145}$-O10/1PN$_{1001}$ 2.9	N/I$_{144}$-O17/1PN$_{1001}$ 3.6	N/A<br>O/D$_{142}$-O19/1PN$_{1001}$ 4.1	N/A$_{279}$-O10/1PN$_{1001}$ 2.7<br>CA/S$_{278}$- O10/1PN$_{1001}$ 3.8 (3.0) 132°
2	4WVJ_A, D	LEP_STAAC	CG/P$_{380_A}$-HOH$_{310}$ 3.6 (2.9) 122°<br>HOH$_{310}$-O/G$_{204_D}$ 4.5<br>O/P$_{380_A}$-CD/P$_{207_D}$ 3.4 (2.5) 144°	N/T$_{382_A}$-O/P$_{207_D}$ 3.0<br>O/T$_{382_A}$-N/A$_{209_D}$ 2.8	N/K$_{384_A}$-O/A$_{209_D}$ 2.9<br>O/K$_{384_A}$-N/A$_{211_D}$ 2.7	N/S$_{387_A}$-O/A$_{211_D}$ 3.0<br>NZ/K$_{428_A}$-O/P$_{212_D}$ 3.2	N/V$_{427_A}$-O/K$_{210_D}$ 2.9	N/D$_{425_A}$-HOH$_{302}$ 2.8<br>HOH$_{302}$-O/T$_{208_D}$ 2.8<br>O/D$_{425_A}$-N/K$_{210_D}$ 2.8	N/K$_{504_A}$-O/P$_{212_D}$ 2.9<br>O/V$_{502_A}$-N/K$_{214_D}$ 2.7
3	4N31_A, E	R9TES9_STRPY	N/V$_{41_A}$-O/F$_{39_E}$ 2.9<br>O/V$_{41_A}$-N/F$_{39_E}$ 3.2	N/I$_{43_A}$-O/Q$_{36_E}$ 2.9<br>O/I$_{43_A}$-N/Q$_{36_E}$ 3.0	N/N$_{45_A}$-O/G$_{34_E}$ 2.8<br>CB/N$_{45_A}$-O/Q$_{33_E}$ 3.3 (2.4) 135°	N/D$_{48_A}$-OG1/T$_{46_A}$ 2.8<br>N/A	N/V$_{84}$-HOH$_{319}$ 3.0<br>HOH$_{319}$-CA/G$_{34}$ 3.3 (2.5) 130°	N/L$_{82_A}$-OE1/Q$_{33_E}$ 3.0<br>O/L$_{82_A}$-N/G$_{34_E}$ 2.7	N/A
					Family: LexA endopeptidase domain-like				
5	8GMU_A	RPC1_LAMBD	N/W$_{142}$-O/P$_{104}$ 2.5<br>O/W$_{142}$-N/F$_{106}$ 2.9	N/E$_{144}$-O/S$_{107}$ 3.6<br>O/E$_{144}$-N/V$_{109}$ 3.1	N/E$_{146}$-O/V$_{109}$ 3.6<br>O/E$_{146}$-N/A$_{111}$ 3.7	N/A<br>N/A	N/F$_{191}$-O/Q$_{110}$ 3.1	N/F$_{189}$-O/S$_{115}$ 3.1<br>O/F$_{189}$-N/S$_{115}$ 3.2	N/A
6	2HNF_A	Q7B004_ECOLX	N/W$_{142}$-O/P$_{104}$ 2.9<br>O/W$_{142}$-N/F$_{106}$ 3.1	N/E$_{144}$-HOH$_{403}$ 2.8<br>HOH$_{403}$-O/F$_{106}$ 2.6<br>O/E$_{144}$-NE2/H$_{108}$ 2.8	N/A	N/A<br>N/A	N/F$_{191}$-HOH$_{406}$ 2.9<br>HOH$_{406}$-O/E$_{117}$ 2.7	N/F$_{189}$-O/R$_{119}$ 2.8<br>O/F$_{189}$-N/R$_{119}$ 2.7	N/A
9	8S7G_A	LEXA_PSEAI	N/L$_{118}$-O/P$_{83}$ 3.9<br>O/L$_{118}$-N/I$_{85}$ 3.6	N/R$_{120}$-O/G$_{86}$ 2.9<br>O/R$_{120}$-N/V$_{88}$ 2.5	N/R$_{122}$-O/V$_{88}$ 3.0<br>O/R$_{122}$-N/A$_{90}$ 2.9	N/A$_{125}$-O/A$_{90}$ 3.4<br>NZ/K$_{162}$-O/G$_{91}$ 2.9	N/V$_{161}$-O/A$_{89}$ 3.2	N/V$_{159}$-O/I$_{94}$ 3.0<br>O/V$_{159}$-N/I$_{94}$ 3.5	N/A
10	1JHE_A	LEXA_ECOLI	N/L$_{112}$-O/P$_{77}$ 2.7<br>O/L$_{112}$-N/V$_{79}$ 3.1	N/R$_{114}$-O/G$_{80}$ 2.8<br>O/R$_{114}$-N/V$_{82}$ 2.9	N/S$_{116}$-O/V$_{82}$ 3.0<br>O/S$_{116}$-N/A$_{84}$ 2.7	N/S$_{119}$-O/A$_{84}$ 3.0<br>CB/A$_{156}$-HOH$_{302}$ 3.6 (2.6) 154°<br>HOH$_{302}$-O/G$_{85}$ 4.1	N/V$_{155}$-O/A$_{83}$ 3.0	N/V$_{153}$-O/L$_{88}$ 2.9<br>O/V$_{153}$-N/L$_{88}$ 3.0	N/A
10a	8GMS_A	LEXA_ECOLI	N/L$_{112}$-O/P$_{77}$ 2.9<br>O/L$_{112}$-N/V$_{79}$ 3.1	N/R$_{114}$-O/G$_{80}$ 2.8<br>O/R$_{114}$-N/V$_{82}$ 3.1	N/S$_{116}$-O/V$_{82}$ 3.0<br>O/S$_{116}$-N/A$_{84}$ 3.1	N/S$_{119}$-O/A$_{84}$ 2.9<br>N/A	N/V$_{155}$-O/A$_{83}$ 3.1	N/V$_{153}$-O/L$_{88}$ 3.1<br>O/V$_{153}$-N/L$_{88}$ 3.3	N/A
10b	8TRG_I	LEXA_ECOLI	N/L$_{112}$-O/P$_{77}$ 3.2<br>O/L$_{112}$-N/V$_{79}$ 3.1	N/R$_{114}$-O/G$_{80}$ 3.0<br>O/R$_{114}$-N/V$_{82}$ 2.7	N/S$_{116}$-O/V$_{82}$ 3.1<br>O/S$_{116}$-N/A$_{84}$ 2.9	N/S$_{119}$-O/A$_{84}$ 4.6<br>N/A	N/V$_{155}$-O/A$_{83}$ 3.3	N/V$_{153}$-O/L$_{88}$ 2.7<br>O/V$_{153}$-N/L$_{88}$ 3.0	N/A
11	8GMT_A, B	UMUD_ECOLI	N/F$_{53_A}$-O/P$_{16_B}$ 3.0<br>O/F$_{53_A}$-N/F$_{18_B}$ 3.8	N/K$_{55_A}$-O/D$_{20_B}$ 5.2<br>O/K$_{55_A}$-N/V$_{22_B}$ 3.2	N/S$_{57_A}$-O/V$_{22_B}$ 2.8<br>O/S$_{57_A}$-N/C$_{24_B}$ 2.4	N/A<br>N/A	N/V$_{96_A}$-O/Q$_{23_B}$ 4.1	N/F$_{94_A}$-O/S$_{28_B}$ 3.7<br>O/F$_{94_A}$-N/S$_{28_B}$ 2.9	N/A
12	2FJR_A	RPC1_BP186	N/A$_{120}$-O/E$_{89}$ 2.8<br>O/A$_{120}$-N/F$_{91}$ 2.8	N/R$_{122}$-O/F$_{91}$ 2.8<br>O/R$_{122}$-N/L$_{93}$ 2.9	N/E$_{124}$-HOH$_{263}$ 2.8<br>HOH$_{263}$-O/L$_{93}$ 2.8<br>N/A	N/A<br>N/A	N/A	N/I$_{149}$-O/E$_{96}$ 3.0<br>O/I$_{149}$-CA/E$_{96}$ 3.2 (2.3) 145°	N/A

N/A–Not Available.

Table S5. Comparison of conservative geometric parameters (distance and angle) in two patterns of the cross-sheet ladder contacts in LexA endopeptidase domain-like family proteins whose 3D structures have been determined by both X-ray diffraction and electron microscopy methods.

N	PDB ID	Protein	I	II	III	IV	V	VI
					Family: LexA endopeptidase domain-like			
5a	1F39_A	RPC1_LAMBD	N/F$_{141}$-O/V$_{168}$ 2.8	N/D$_{169}$-O/K$_{224}$ 2.8	N/G$_{223}$-O/I$_{181}$ 3.4	N/A$_{182}$-O/T$_{190}$ 2.8	N/A	N/A
			O/F$_{141}$-N/V$_{168}$ 2.8	O/D$_{169}$-N/K$_{224}$ 3.1	O/G$_{223}$-N/I$_{181}$ 3.0	O/A$_{182}$-N/T$_{190}$ 3.0		
5b			N/L$_{143}$-O/I$_{166}$ 2.9	N/L$_{167}$-O/A$_{227}$ 2.8	N/V$_{225}$-O/F$_{179}$ 3.0	N/C$_{180}$-O/K$_{192}$ 2.9	N/K$_{193}$-O/Q$_{204}$ 2.8	N/L$_{203}$-O/I$_{213}$ 2.7
	≡	≡	O/L$_{143}$-N/I$_{166}$ 2.9	O/L$_{167}$-N/I$_{226}$ 2.8	O/V$_{225}$-HOH$_{31}$ 2.9	O/C$_{180}$-N/K$_{192}$ 3.1	O/K$_{193}$N/Q$_{204}$ 2.7	O/L$_{203}$-N/I$_{213}$ 2.9
					HOH$_{31}$-N/F$_{179}$ 2.8			
5aA	8GMU_A	RPC1_LAMBD	N/F$_{141}$-O/V$_{168}$ 3.0	N/D$_{169}$-O/K$_{224}$ 2.9	N/G$_{223}$-O/I$_{181}$ 3.5	N/A$_{182}$-O/T$_{190}$ 3.0	N/A	N/A
			O/F$_{141}$-N/V$_{168}$ 2.8	O/D$_{169}$-N/K$_{224}$ 3.0	O/G$_{223}$-N/I$_{181}$ 3.0	O/A$_{182}$-N/T$_{190}$ 3.0		
5bA	≡	≡	N/L$_{143}$-O/I$_{166}$ 2.8	N/L$_{167}$-O/A$_{227}$ 3.1	N/V$_{225}$-O/F$_{179}$ 2.8	N/C$_{180}$-O/K$_{192}$ 3.0	N/K$_{193}$-O/Q$_{204}$ 3.4	N/L$_{203}$-O/I$_{213}$ 3.0
			O/L$_{143}$-N/I$_{166}$ 2.6	O/L$_{167}$-N/I$_{226}$ 2.9	O/V$_{225}$-N/F$_{179}$ 4.9	O/C$_{180}$-N/K$_{192}$ 3.3	O/K$_{193}$N/Q$_{204}$ 3.1	O/L$_{203}$-N/I$_{213}$ 2.8
8a	8B0V_A	LEXA_PSEAI	N/Y$_{117}$-O/V$_{139}$ 3.4	N/H$_{140}$-O/L$_{197}$ 2.9	N/E$_{195}$-O/V$_{152}$ 2.8	N/A$_{153}$-O/T$_{160}$ 2.9	N/A	N/A
			O/Y$_{117}$-N/V$_{139}$ 2.9	O/H$_{140}$-N/L$_{197}$ 2.9	O/G$_{196}$-N/V$_{152}$ 2.9	O/A$_{153}$-N/T$_{160}$ 2.9		
8b	≡	≡	N/L$_{119}$-O/L$_{137}$ 2.8	N/A$_{138}$-O/G$_{200}$ 2.9	N/S$_{198}$-O/V$_{150}$ 2.9	N/V$_{151}$-O/K$_{162}$ 2.9	N/R$_{163}$-O/L$_{174}$ 2.9	N/L$_{173}$-O/I$_{183}$ 2.7
			O/L$_{119}$-N/L$_{137}$ 2.8	O/A$_{138}$-N/V$_{199}$ 2.8	O/S$_{198}$-HOH$_{415}$ 4.9	O/V$_{151}$-N/K$_{162}$ 3.1	O/R$_{163}$-N/L$_{174}$ 2.8	O/L$_{173}$-N/I$_{183}$ 3.0
					HOH$_{415}$-N/V$_{150}$ 3.0			
8aA	8S7G_A	LEXA_PSEAI	N/Y$_{117}$-O/V$_{139}$ 3.6	N/H$_{140}$-O/L$_{197}$ 2.9	N/E$_{195}$-O/V$_{152}$ 2.7	N/A$_{153}$-O/T$_{160}$ 2.7	N/A	N/A
			O/Y$_{117}$-N/V$_{139}$ 2.9	O/H$_{140}$-N/L$_{197}$ 3.1	O/G$_{196}$-N/V$_{152}$ 2.7	O/A$_{153}$-N/T$_{160}$ 2.8		
8bA	≡	≡	N/L$_{119}$-O/L$_{137}$ 2.5	N/A$_{138}$-O/G$_{200}$ 3.0	N/S$_{198}$-O/V$_{150}$ 3.4	N/V$_{151}$-O/K$_{162}$ 3.0	N/R$_{163}$-O/L$_{174}$ 3.0	N/L$_{173}$-O/I$_{183}$ 2.7
			O/L$_{119}$-N/L$_{137}$ 3.0	O/A$_{138}$-N/V$_{199}$ 3.2	OG/S$_{198}$-O/V$_{150}$ 5.7	O/V$_{151}$-N/K$_{162}$ 3.2	O/R$_{163}$-N/L$_{174}$ 3.2	O/L$_{173}$-N/I$_{183}$ 3.3
9a	1JHF_A	LEXA_ECOLI	N/F$_{111}$-O/V$_{133}$ 3.5	N/H$_{134}$-O/L$_{191}$ 2.9	N/G$_{190}$-O/V$_{146}$ 3.4	N/A$_{147}$-O/T$_{154}$ 3.0	N/A	N/A
			O/F$_{111}$-N/V$_{133}$ 2.9	O/H$_{134}$-N/L$_{191}$ 3.0	O/G$_{190}$-N/V$_{146}$ 3.0	O/A$_{147}$-N/T$_{154}$ 2.9		
9b			N/L$_{113}$-O/L$_{131}$ 2.9	N/A$_{132}$-O/G$_{194}$ 2.9	N/A$_{192}$-O/V$_{144}$ 3.2	N/V$_{145}$-O/K$_{156}$ 2.9	N/R$_{157}$-O/L$_{168}$ 3.0	N/L$_{167}$-O/I$_{177}$ 2.9
	≡	≡	O/L$_{113}$-N/L$_{131}$ 2.9	O/A$_{132}$-N/V$_{193}$ 2.9	O/A$_{192}$-HOH$_{433}$ 2.7	O/V$_{145}$-N/K$_{156}$ 3.2	O/R$_{157}$-N/L$_{168}$ 2.9	O/L$_{167}$-N/I$_{177}$ 3.1
					HOH$_{433}$-N/V$_{144}$ 3.4			
9aA	1JHE_A	LEXA_ECOLI	N/F$_{111}$-O/V$_{133}$ 3.3	N/H$_{134}$-O/L$_{191}$ 3.0	N/G$_{190}$-O/V$_{146}$ 3.5	N/A$_{147}$-O/T$_{154}$ 3.0	N/A	N/A
			O/F$_{111}$-N/V$_{133}$ 3.0	O/H$_{134}$-N/L$_{191}$ 3.1	O/G$_{190}$-N/V$_{146}$ 3.0	O/A$_{147}$-N/T$_{154}$ 3.1		
9bA	≡	≡	N/L$_{113}$-O/L$_{131}$ 3.1	N/A$_{132}$-O/G$_{194}$ 2.8	N/A$_{192}$-O/V$_{144}$ 3.0	N/V$_{145}$-O/K$_{156}$ 2.8	N/R$_{157}$-O/L$_{168}$ 2.8	N/L$_{167}$-O/I$_{177}$ 2.8
			O/L$_{113}$-N/L$_{131}$ 2.9	O/A$_{132}$-N/V$_{193}$ 3.2	O/A$_{192}$-N/V$_{144}$ 5.4	O/V$_{145}$-N/K$_{156}$ 3.2	O/R$_{157}$-N/L$_{168}$ 2.8	O/L$_{167}$-N/I$_{177}$ 2.9
9aB	8GMS_A	LEXA_ECOLI	N/F$_{111}$-O/V$_{133}$ 3.0	N/H$_{134}$-O/L$_{191}$ 3.0	N/G$_{190}$-O/V$_{146}$ 3.6	N/A$_{147}$-O/T$_{154}$ 2.9	N/A	N/A
			O/F$_{111}$-N/V$_{133}$ 2.9	O/H$_{134}$-N/L$_{191}$ 3.0	O/G$_{190}$-N/V$_{146}$ 3.5	O/A$_{147}$-N/T$_{154}$ 2.9		
9bB	≡	≡	N/L$_{113}$-O/L$_{131}$ 2.9	N/A$_{132}$-O/G$_{194}$ 2.9	N/A$_{192}$-O/V$_{144}$ 3.0	N/V$_{145}$-O/K$_{156}$ 2.9	N/R$_{157}$-O/L$_{168}$ 2.9	N/L$_{167}$-O/I$_{177}$ 3.1
			O/L$_{113}$-N/L$_{131}$ 2.9	O/A$_{132}$-N/V$_{193}$ 3.0	O/A$_{192}$-N/V$_{144}$ 5.7	O/V$_{145}$-N/K$_{156}$ 3.0	O/R$_{157}$-N/L$_{168}$ 2.9	O/L$_{167}$-N/I$_{177}$ 3.7
9aC	8TRG _I	LEXA_ECOLI	N/F$_{111}$-O/V$_{133}$ 3.5	N/H$_{134}$-O/L$_{191}$ 2.8	N/G$_{190}$-O/V$_{146}$ 3.8	N/A$_{147}$-O/T$_{154}$ 3.1	N/A	N/A
			O/F$_{111}$-N/V$_{133}$ 3.0	O/H$_{134}$-N/L$_{191}$ 3.0	O/G$_{190}$-N/V$_{146}$ 3.6	O/A$_{147}$-N/T$_{154}$ 3.1		
9bC	≡	≡	N/L$_{113}$-O/L$_{131}$ 3.0	N/A$_{132}$-O/G$_{194}$ 3.3	N/A$_{192}$-O/V$_{144}$ 3.5	N/V$_{145}$-O/K$_{156}$ 3.1	N/R$_{157}$-O/L$_{168}$ 3.6	N/L$_{167}$-O/I$_{177}$ 2.9
			O/L$_{113}$-N/L$_{131}$ 2.8	O/A$_{132}$-N/V$_{193}$ 2.9	O/A$_{192}$-N/V$_{144}$ 6.2	O/V$_{145}$-N/K$_{156}$ 3.4	O/R$_{157}$-N/L$_{168}$ 3.2	O/L$_{167}$-N/I$_{177}$ 3.1

11a	1UMU_A	UMUD_ECOLI	N/Y_{52}-O/V_{74} 2.9<br>O/Y_{52}-N/V_{74} 2.7	N/D_{75}-O/V_{130} 3.0<br>O/D_{75}-N/V_{130} 3.1	N/G_{129}-O/I_{87} 2.8<br>O/G_{129}-N/I_{87} 3.2	N/A_{88}-O/T_{95} 2.8<br>O/A_{88}-N/T_{95} 2.7	N/A	N/A
11b	≡	≡	N/V_{54}-O/L_{72} 3.0<br>O/V_{54}-N/L_{72} 2.7	N/I_{73}-O/H_{133} 2.9<br>O/I_{73}-N/I_{132} 2.8	N/V_{131}-O/I_{85} 2.8<br>O/V_{131}-HOH_{141} 2.8<br>HOH_{141}-N/I_{85} 2.8	N/V_{86}-O/K_{97} 2.8<br>O/V_{86}-N/K_{97} 3.0	N/K_{98}-O/I_{108} 3.1<br>O/K_{98}-N/I_{108} 3.0	N/L_{107}-O/I_{117} 2.7<br>O/L_{107}-N/I_{117} 2.9
11aA	8GMT_A	UMUD_ECOLI	N/Y_{52}-O/V_{74} 3.2<br>O/Y_{52}-N/V_{74} 3.0	N/D_{75}-O/V_{130} 3.0<br>O/D_{75}-N/V_{130} 3.1	N/G_{129}-O/I_{87} 3.9<br>O/G_{129}-N/I_{87} 3.1	N/A_{88}-O/T_{95} 2.7<br>O/A_{88}-N/T_{95} 2.4	N/A	N/A
11bA	≡	≡	N/V_{54}-O/L_{72} 2.7<br>O/V_{54}-N/L_{72} 2.5	N/I_{73}-O/H_{133} 3.0<br>O/I_{73}-N/I_{132} 2.9	N/V_{131}-O/I_{85} 2.7<br>O/V_{131}-N/I_{85} 5.1	N/V_{86}-O/K_{97} 3.2<br>O/V_{86}-N/K_{97} 3.3	N/K_{98}-O/I_{108} 2.9<br>O/K_{98}-N/I_{108} 3.0	N/L_{107}-O/I_{117} 2.5<br>O/L_{107}-N/I_{117} 2.6

N/A–Not Available.

Table S6. Comparison of conservative geometric parameters (distance and angle) of the Omega and NucBaseOmega subzones in LexA endopeptidase domain-like family proteins whose 3D structures have been determined by both X-ray diffraction and electron microscopy methods.

N	PDB ID	Protein	VII	VIII	IX	X	XI
			Family: LexA endopeptidase domain-like				
5	1F39_A	RPC1_LAMBD	CG/P$_{205}$-O/P$_{211}$ 3.5 (2.7) 132°	O/N$_{207}$-N/Y$_{210}$ 2.9 OD1/N$_{207}$-N/Q$_{209}$ 3.0	O/S$_{149}$-N/N$_{207}$ 3.1 O/N$_{148}$-ND2/N$_{207}$ 2.9	CB/K$_{192}$-O/Q$_{204}$ 3.7 (2.7) 142°	OG/S$_{149}$-NZ/K$_{192}$ 3.1
5A	8GMU_A	RPC1_LAMBD	CG/P$_{205}$-O/P$_{211}$ 3.3 (2.7) 113°	O/N$_{207}$-N/Y$_{210}$ 3.2 N/A	O/S$_{149}$-N/N$_{207}$ 3.0 O/N$_{148}$-ND2/N$_{207}$ 3.0	CB/A$_{192}$-O/Q$_{204}$ 4.0 (2.9) 161°	OG/S$_{149}$-CB/A$_{192}$ 6.8 (6.0) 142°
8	8B0V_A	LEXA_PSEAI	CB/A$_{175}$-HOH$_{416}$ 3.7 (2.8) 138° HOH$_{416}$-O/A$_{181}$ 2.9	O/N$_{177}$-N/F$_{180}$ 3.0 OD1/N$_{177}$-N/E$_{179}$ 3.0	O/S$_{125}$-N/E$_{176}$ 2.8 O/M$_{124}$-ND2/N$_{177}$ 2.8	CB/K$_{162}$-O/L$_{174}$ 3.6 (2.9) 124°	OG/S$_{125}$-NZ/K$_{162}$ 2.8
8A	8S7G_A	LEXA_PSEAI	CB/A$_{175}$-O/A$_{181}$ 5.3 (4.6) 120°	O/N$_{177}$-N/F$_{180}$ 3.2 N/A	O/A$_{125}$-N/E$_{176}$ 3.5 O/M$_{124}$-ND2/N$_{177}$ 2.7	CB/K$_{162}$-O/L$_{174}$ 3.6 (2.7) 132°	CB/A$_{125}$-CE/K$_{162}$ 3.6
9	1JHF_A	LEXA_ECOLI	CG/P$_{169}$-O/K$_{175}$ 4.1 (3.2) 137°	O/N$_{171}$-N/F$_{174}$ 3.1 OD1/N$_{171}$-N/E$_{173}$ 2.9	O/S$_{119}$-N/E$_{170}$ 2.8 O/M$_{118}$-ND2/N$_{171}$ 2.9	CB/K$_{156}$-O/L$_{168}$ 3.7 (3.0) 121°	OG/S$_{119}$-NZ/K$_{156}$ 2.9
9A	1JHE_A	LEXA_ECOLI	CG/P$_{169}$-O/K$_{175}$ 4.0 (3.1) 135°	O/N$_{171}$-N/F$_{174}$ 2.9 OD1/N$_{171}$-N/E$_{173}$ 2.9	O/S$_{119}$-N/E$_{170}$ 2.7 O/M$_{118}$-ND2/N$_{171}$ 2.9	CB/A$_{156}$-O/L$_{168}$ 2.9 (1.9) 153°	OG/S$_{119}$-HOH$_{302}$ 2.6 HOH$_{302}$-CB/A$_{156}$ 3.6 (2.6) 154°
9B	8GMS_A	LEXA_ECOLI	CG/P$_{169}$-O/K$_{175}$ 3.8 (3.0) 136°	O/N$_{171}$-N/F$_{174}$ 3.7 OD1/N$_{171}$-N/E$_{173}$ 2.5	O/S$_{119}$-N/E$_{170}$ 2.6 O/M$_{118}$-ND2/N$_{171}$ 3.0	CB/A$_{156}$-O/L$_{168}$ 3.2 (2.2) 155°	OG/S$_{119}$-CB/A$_{156}$ 5.8 (4.9) 142°
9C	8TRG _I	LEXA_ECOLI	CG/P$_{169}$-O/K$_{175}$ 4.1 (3.2) 137°	O/N$_{171}$-N/F$_{174}$ 3.1 OD1/N$_{171}$-N/E$_{173}$ 3.1	O/S$_{119}$-N/E$_{170}$ 3.0 O/M$_{118}$-ND2/N$_{171}$ 3.1	CB/A$_{156}$-O/L$_{168}$ 3.3 (2.3) 150°	OG/S$_{119}$-CB/A$_{156}$ 6.6 (5.9) 131°
11	1UMU_A	UMUD_ECOLI	CG/P$_{109}$-O/S$_{115}$ 4.2 (3.5) 127°	O/N$_{111}$-N/Y$_{114}$ 2.9 OD1/N$_{111}$-N/A$_{113}$ 2.8	O/S$_{60}$-N/M$_{110}$ 2.7 O/D$_{59}$-ND2/N$_{111}$ 2.7	CB/K$_{97}$-O/I$_{108}$ 3.9 (3.0) 135°	OG/S$_{60}$-NZ/K$_{97}$ 2.8
11A	8GMT_A	UMUD_ECOLI	CG/P$_{109}$-O/S$_{115}$ 3.7 (2.9) 126°	O/N$_{111}$-N/Y$_{114}$ 3.2 OD1/N$_{111}$-N/A$_{113}$ 2.9	O/S$_{60}$-N/M$_{110}$ 2.9 O/D$_{59}$-ND2/N$_{111}$ 2.6	CB/A$_{97}$-O/I$_{108}$ 3.9 (3.0) 137°	OG/S$_{60}$-CB/A$_{97}$ 6.9 (6.2) 126°

N/A–Not Available.
